# Species Diversity of *Helvella lacunosa* Clade (Pezizales, Ascomycota) in China and Description of Sixteen New Species

**DOI:** 10.3390/jof9070697

**Published:** 2023-06-23

**Authors:** Xin-Cun Wang, Wen-Ying Zhuang, Rui-Lin Zhao

**Affiliations:** State Key Laboratory of Mycology, Institute of Microbiology, Chinese Academy of Sciences, Beijing 100101, China; wangxc@im.ac.cn (X.-C.W.); zhaorl@im.ac.cn (R.-L.Z.)

**Keywords:** Ascomycota, biodiversity, Helvellaceae, new species, phylogeny, taxonomy

## Abstract

*Helvella lacunosa* and its allies are widely distributed in the Northern Hemisphere and perform important functions in ecosystems. A comprehensive study on 101 collections of *Helvella lacunosa*, including those deposited in four Chinese fungaria or collected recently from 10 provinces, was conducted based on morphological and molecular characteristics. Phylogenies of “*Helvella lacunosa* clade” inferred from Hsp90, ITS, LSU, and TEF were reconstructed with 49 lineages recognized, of which 25 lineages occurred in China, and each represented an individual species. Sixteen new species were determined with detailed descriptions and illustrations. Two new Chinese records were reported. Species concepts and their distinctions in macro- and micro-features were discussed.

## 1. Introduction

The *Helvella lacunosa* species complex (Helvellaceae, Pezizales) contains ascomycetous macrofungi having dark, saddle-shaped, lobed, or convex apothecia with sulcate or ribbed stipes. They are widely distributed in the Northern Hemisphere and perform important functions in ecosystems [1,2]. Some *Helvella* species are taken as precious and delicious food by local people in Shanxi and Xinjiang provinces of China.

The *Helvella* section *Lacunosae* was established by Dissing [3] to accommodate a group of operculate cup-fungi, including *H. lactea* Boud., *H. lacunosa* Afzel., *H. philonotis* Dissing, and *H. phlebophora* Pat. & Doass. The section was accepted by Weber [4] and Häffner [5] with *H. palustris* Peck and *H. sulcata* Afzel. added, which were treated as synonyms of *H. lacunosa* by Dissing [3]. Morphological features of the section include epigeous, stipitate, saddle-shaped or lobed apothecia; ribbed or lacunose stipes; a usually dark-colored hymenium surface; subcylindrical asci; filiform or clavate paraphyses; and ellipsoidal, hyaline, smooth, uniguttulate ascospores. Fragments of small subunit (SSU) and large subunit (LSU) ribosomal RNA genes were first adopted for pezizalean phylogenetic analyses in the 1990s [6] and were followed by subsequent studies [7,8,9,10,11,12,13]. Translation elongation factor 1-α (TEF) and other protein-coding genes were also applied to infer the phylogeny of this group of fungi [10,14,15,16,17]. As to *Helvella*, the internal transcribed spacer (ITS) and LSU phylogenies of *H. lacunosa* species complex were initially reported by Nguyen et al. [18]. And then, phylogenies of the genus based on a single gene, ITS or LSU, were reconstructed [2,19]. Hsp90 was introduced into the analysis of the genus and advocated as a primary DNA barcode [20]. Up to now, LSU is still the most popular locus to be used for species identification of the group, although its capacity is limited. Sequences of Hsp90 and ITS are usually lacking for some *Helvella* species, which creates barriers for species recognition. Our previous study [21] on the rib-stiped cupulate species of the genus tried to address this aspect.

Through the aid of molecular phylogenetics, species concepts of *Helvella* became well-established. A synopsis of the European species was accomplished by Skrede et al. [20], in which the monophyletic Clade C corresponded to the section containing *H. lacunosa* and its allies (as *Helvella lacunosa* clade). A total of 22 lineages were revealed in the clade based on multiple sequence analyses, and epi-/neo-types were designated for *H. atra* Oeder, *H. helvellula* (Durieu & Mont.) Dissing, *H. lacunosa*, *H. nigra* Bergeret, *H. pallescens* Schaeff., *H. phlebophora*, *H. queletiana* Sacc. & Traverso, and *H. sulcata*. Thereafter, nine additional species of *Helvella* were described from Spain [22], one from Mexico [23], and one from China [24], among which nine species belonged to this clade: *H. cystidiata* R.J. Xu et al., *H. fuscolacunosa* Skrede & T. Schumach., *H. hispanica* Skrede & T. Schumach., *H. iberica* Skrede & T. Schumach., *H. inexpectata* Skrede & T. Schumach., *H. jocatoi* F. Landeros et al., *H. neopallescens* Skrede & T. Schumach., *H. phlebophoroides* Skrede & T. Schumach., and *H. terricola* Skrede & T. Schumach.

In China, *H. lacunosa* was first recorded by Teng [25] from seven provinces, and then *H. atra*, *H. lacunosa*, *H. pallescens,* and *H. lacteal* were listed by Tai [26]. More taxa of the clade were subsequently reported: *H. helvellula*, *H. lactea,* and *H. phlebophora* by Liu and Cao [27]; *H. philonotis* by Zhuang and Wang [28]; and *H. fusca* Gillet by Xu [29]. Recently, *H. cystidiata*, *H. pseudolacunosa* Q. Zhao & K.D. Hyde, *H. rugosa* Q. Zhao & K.D. Hyde, and *H. sublactea* Q. Zhao et al. were further discovered and described based on the Chinese materials [24,30,31]. A total of 13 species of the group were known in the country.

In connection with our current work on the family Helvellaceae of Flora Fungorum Sinicorum, extensive surveys of *Helvella* collections from different regions of China have been conducted. The results of a study on the species possessing cupulate apothecia with sulcate or ribbed stipes were previously reviewed [15]. In this work, members of of *Helvella lacunosa* clade were investigated, and it turned out that species diversity of the clade is extremely high. The current research is a continuation towards the classification of the group based on multigene analysis.

## 2. Materials and Methods

### 2.1. Re-Examination and Collection of Samples

Collections of the *Helvella lacunosa* clade from China deposited in the following fungaria were re-examined: Herbarium Mycologicum Academiae Sinicae (HMAS), Herbarium of the Microbiology Institute of Guangdong (HMIGD), Herbarium of Mycological Institute of Jilin Agricultural University (HMJAU), and Mycological Herbarium of Chifeng University (CFSZ). Specimens recently collected from 10 cities, provinces, or administrative regions of China (Beijing, Fujian, Guizhou, Jiangsu, Jilin, Shanxi, Sichuan, Tibet, Yunnan, and Zhejiang) were also identified (Table 1). Morphological observations were conducted following the methods indicated in previous studies [21,32].

### 2.2. DNA Extraction, PCR Amplification and Sequencing

Well-preserved specimens were selected for DNA extraction using a Plant Genomic DNA Kit (DP305, TIANGEN Biotech, Beijing, China). Portions of the heat shock protein 90 (Hsp90), nuclear ribosomal DNA internal transcribed spacer (ITS), large subunit (LSU), and translation elongation factor 1-α (TEF) genes were amplified via PCR using the primer pairs H_hspf and H_hspr [20], ITS5 (or ITS3) and ITS4 [33], LROR and LR5 [34], and EF1-983F and EF1-1567R [35]. Products were purified and sequenced on an ABI 3730 DNA Sequencer (Applied Biosystems, Bedford, MA, USA).

### 2.3. Phylogenetic Analyses

In this study, newly generated forward and reverse sequences were assembled using Seqman v. 7.1.0 (DNASTAR Inc., Madison, WI, USA). The assembled sequences and those retrieved from GenBank are listed in Table 1. Four single-gene datasets and a multi-locus dataset were compiled. Sequences were aligned using MAFFT v. 7.221 [36] and then manually edited via BioEdit v. 7.1.10 [37] and MEGA v. 6.0.6 [38]. Maximum likelihood (ML) analyses were performed using RAxML-HPC2 [39] on XSEDE 8.2.12 on CIPRES Science Gateway v. 3.3 [40] with the default GTRCAT model. Bayesian inference (BI) analyses were performed with MrBayes v. 3.2.5 [41]. Appropriate nucleotide substitution models and parameters were determined via Modeltest v. 3.7 [42]. The consensus trees were viewed in FigTree v. 1.3.1 (http://tree.bio.ed.ac.uk/software/figtree/, accessed on 1 September 2015). Two species of *Dissingia* served as outgroup taxa.

**Table 1:** Fungal species and sequences used in phylogenetic analyses.

Species	Voucher	Origin	Hsp90	ITS	LSU	TEF	Reference
*Helvella atra* Oeder 1770	O-255762 = H1055	Norway	MN692348	MN656170	MN655852	MN689304	[43]
	10865 = HMAS 290900	China: Tibet	**OQ597592**	**OQ600286**	**OQ586677**	**OQ597533**	This study
	11222 = HMAS 290901	China: Tibet	**OQ597593**	**OQ600287**	**OQ586678**	**OQ597534**	This study
	HMAS 83545	China: Xinjiang	**OQ597594**	n.a.	n.a.	**OQ597535**	This study
	HMAS 265533	China: Tibet	**OQ597595**	**OQ600288**	n.a.	**OQ597536**	This study
	HMJAU 27662	China: Inner Mongolia	**OQ597596**	**OQ600289**	**OQ586679**	n.a.	This study
***H. austrooccidentalis*** X.C. Wang & W.Y. Zhuang, sp. nov.	11220 = HMAS 290902	China: Tibet	**OQ597597**	**OQ600290**	**OQ586680**	**OQ597537**	This study
	11223 = HMAS 290903, holotype	China: Tibet	**OQ597598**	**OQ600291**	**OQ586681**	**OQ597538**	This study
	ZRL20200655 = HMAS 290904	China: Sichuan	**OQ597599**	**OQ600292**	**OQ586682**	n.a.	This study
***H. borealis*** X.C. Wang & W.Y. Zhuang, sp. nov.	3568 = HMAS 290905, holotype	China: Jilin	**OQ597600**	**OQ600293**	**OQ586683**	**OQ597539**	This study
	UC 1999199	USA: Minnesota	n.a.	n.a.	KC122796	n.a.	[18]
*H. cystidiata* R.J. Xu et al. 2022	HKAS 78941, holotype	China: Yunnan	n.a.	KX239839	KX239802	n.a.	[24]
	HKAS 74316	China: Yunnan	n.a.	KX239840	KX239803	n.a.	[24]
	HMAS 275836	China: Yunnan	**OQ597601**	**OQ600294**	n.a.	n.a.	This study
	HMJAU 52	China: Tibet	**OQ597602**	**OQ600295**	**OQ586684**	**OQ597540**	This study
	HMJAU 150	China: Tibet	**OQ597603**	**OQ600296**	**OQ586685**	**OQ597541**	This study
*H. dryophila* Vellinga & N.H. Nguyen 2013	UC 1999238 = MES218	USA: California	n.a.	KC122811	KC122772	n.a.	[18]
***H. fulva*** X.C. Wang & W.Y. Zhuang, sp. nov.	10867 = HMAS 290906, holotype	China: Tibet	**OQ597604**	**OQ600297**	**OQ586686**	**OQ597542**	This study
*H. fusca* Gillet 1879	C-F-92122 = H305	Hungary	KY784415	n.a.	KY773101	n.a.	[20]
*H. fuscolacunosa* Skrede & T. Schumach. 2020	TRH12618 = H2883, holotype	Spain	MN598173	n.a.	MN644495	n.a.	[22]
*H. helvellula* (Durieu & Mont.) Dissing 1966	C-F-45507 = H278	France	KY784393	n.a.	KY773090	n.a.	[20]
*H. hispanica* Skrede & T. Schumach. 2020	O-F-256536 = H1023	Spain	MN598112	n.a.	MN644504	n.a.	[22]
***H. huangii*** X.C. Wang & W.Y. Zhuang, sp. nov.	1414 = HMAS 290907	China: Beijing	**OQ597605**	**OQ600298**	**OQ586687**	**OQ597543**	This study
	HMAS 45031, holotype	China: Beijing	**OQ597606**	**OQ600299**	**OQ586688**	n.a.	This study
	HMJAU 3488	China: Jilin	**OQ597607**	**OQ600300**	**OQ586689**	**OQ597544**	This study
	CFSZ 2652	China: Inner Mongolia	**OQ597608**	n.a.	n.a.	n.a.	This study
*H. iberica* Skrede & T. Schumach. 2020	O-F256539 = H1018, holotype	Spain	MN598109	n.a.	MN644498	n.a.	[22]
*H. inexpectata* Skrede & T. Schumach. 2020	O-F-256540 = H1017	Spain	MN598192	n.a.	n.a.	n.a.	[22]
***H. jizushanica*** X.C. Wang & W.Y. Zhuang, sp. nov.	11567 = HMAS 290908, holotype	China: Yunnan	**OQ597609**	**OQ600301**	**OQ586690**	**OQ597545**	This study
	HMAS 59718	China: Yunnan	**OQ597610**	**OQ600302**	**OQ586691**	n.a.	This study
*H. jocatoi* F. Landeros et al. 2021	CB08326 = MEXU 25760, holotype	Mexico	n.a.	KC016115	MH399851	n.a.	[18,23]
*H. juniperi* M. Filippa & Baiano 1999	H2973, holotype	Italy	MN598194	n.a.	n.a.	n.a.	[22]
*H. lactea* Boud. 1907	C-F-39379 = H374	Denmark	KY784473	n.a.	n.a.	n.a.	[20]
	CUP 52755, isotype of *H. astieri* Korf and Donadini	France	MK238676	n.a.	MK129270	n.a.	[44]
*H. lacunosa* Afzel. 1783	H407, epitype	Sweden	KY784503	n.a.	KY773152	n.a.	[20]
	O-255761 = H1041	Norway	MN692347	MN656169	MN655855	MN689302	[43]
	HKAS 87594, holotype of *H. pseudolacunosa* Q. Zhao and K.D. Hyde	China: Inner Mongolia	n.a.	KR493476	KT932629	n.a.	[30,45]
	HMAS 57959	China: Shanxi	**OQ597611**	n.a.	n.a.	n.a.	This study
	HMAS 61369	China: Shanxi	**OQ597612**	**OQ600303**	**OQ586692**	n.a.	This study
	HMAS 83536	China: Xinjiang	**OQ597613**	**OQ600304**	n.a.	**OQ597546**	This study
	HMAS 85622	China: Shanxi	**OQ597614**	**OQ600305**	n.a.	n.a.	This study
	HMAS 86534	China: Shanxi	**OQ597615**	**OQ600306**	**OQ586693**	**OQ597547**	This study
	HMAS 98364	China: Shanxi	**OQ597616**	n.a.	n.a.	n.a.	This study
	HMJAU 6816	China: Inner Mongolia	**OQ597617**	**OQ600307**	**OQ586694**	**OQ597548**	This study
	HMJAU 6817	China: Inner Mongolia	**OQ597618**	**OQ600308**	**OQ586695**	**OQ597549**	This study
	HMJAU 22359	Belarus	**OQ597619**	n.a.	n.a.	n.a.	This study
	HMJAU 23240	China: Inner Mongolia	**OQ597620**	**OQ600309**	**OQ586696**	**OQ597550**	This study
	ChenJQ 01 = HMAS 290909	China: Shanxi	**OQ597621**	n.a.	**OQ586697**	**OQ597551**	This study
***H. liui*** X.C. Wang & W.Y. Zhuang, sp. nov.	HMAS 85725, holotype	China: Shanxi	**OQ597622**	**OQ600310**	n.a.	n.a.	This study
	XT13106	China: Hebei	n.a.	MF405782	n.a.	n.a.	[46]
***H. lobata*** X.C. Wang & W.Y. Zhuang, sp. nov.	HaiY01 = HMAS 290910, holotype	China: Jiangsu	**OQ597623**	**OQ600311**	**OQ586698**	n.a.	This study
***H. magna*** X.C. Wang & W.Y. Zhuang, sp. nov.	10861 = HMAS 290911	China: Tibet	**OQ597624**	**OQ600312**	**OQ586699**	**OQ597552**	This study
	10864 = HMAS 290912	China: Tibet	**OQ597625**	**OQ600313**	**OQ586700**	**OQ597553**	This study
	11790 = HMAS 290913	China: Yunnan	**OQ597626**	**OQ600314**	**OQ586701**	**OQ597554**	This study
	HMAS 60679, holotype	China: Gansu	**OQ597627**	**OQ600315**	**OQ586702**	**OQ597555**	This study
	HMAS 61724	China: Gansu	**OQ597628**	n.a.	**OQ586703**	n.a.	This study
	HMAS 66121	China: Gansu	**OQ597629**	**OQ600316**	**OQ586704**	**OQ597556**	This study
	HMAS 69594	China: Gansu	**OQ597630**	n.a.	n.a.	n.a.	This study
	HMAS 69595	China: Gansu	**OQ597631**	**OQ600317**	**OQ586705**	n.a.	This study
	HMAS 70345	China: Beijing	**OQ597632**	**OQ600318**	**OQ586706**	n.a.	This study
	HMAS 75848	China: Beijing	**OQ597633**	**OQ600319**	n.a.	n.a.	This study
*H. neopallescens* Skrede & T. Schumach. 2020	O-F-256550 = H1022, holotype	Spain	MN598111	n.a.	MN644500	n.a.	[22]
*H. nigra* Bergeret 1783	O-253345 = H063, epitype	Sweden	KY784227	n.a.	KY772947	KY772855	[20]
	UC 1999221	USA: New Hampshire	n.a.	KC122819	n.a.	n.a.	[18]
	HMAS 58374	Denmark	**OQ597634**	n.a.	n.a.	n.a.	This study
	HMAS 244000	UK	**OQ597635**	**OQ600320**	**OQ586707**	**OQ597557**	This study
	HMAS 262949	Italy	**OQ597636**	**OQ600321**	**OQ586708**	**OQ597558**	This study
*H. pallescens* Schaeff. 1774	O-66205 = H138, epitype	Norway	KY784271	n.a.	KY772988	KY772878	[20]
	HMAS 243999	UK	**OQ597637**	**OQ600322**	**OQ586709**	**OQ597559**	This study
*H. palustris* Peck 1883	O-253359 = H043	Norway	KY784214	n.a.	KY772933	KY772848	[20]
	HMAS 30755	China: Jilin	**OQ597638**	n.a.	n.a.	n.a.	This study
***H. parva*** X.C. Wang & W.Y. Zhuang, sp. nov.	11559 = HMAS 290914, holotype	China: Yunnan	**OQ597639**	**OQ600323**	**OQ586710**	**OQ597560**	This study
*H. philonotis* Dissing 1964	O-255760 = H2110	Norway	MN692353	MN656182	MN655853	MN689303	[43]
	10695 = HMAS 290915	China: Tibet	**OQ597640**	**OQ600324**	**OQ586711**	**OQ597561**	This study
	HMAS 30756	China: Qinghai	**OQ597641**	n.a.	n.a.	n.a.	This study
	HMAS 51197	China: Sichuan	**OQ597642**	**OQ600325**	n.a.	n.a.	This study
	HMAS 262553	China: Tibet	**OQ597643**	**OQ600326**	n.a.	**OQ597562**	This study
	HMAS 264754	China: Tibet	**OQ597644**	**OQ600327**	**OQ586712**	**OQ597563**	This study
*H. phlebophora* Pat. & Doass. 1886	C-F-45405 = H273	Iceland	KY784388	n.a.	KY773087	n.a.	[20]
	HMAS 268001	China: Qinghai	**OQ597645**	**OQ600328**	**OQ586713**	**OQ597564**	This study
*H. phlebophoroides* Skrede & T. Schumach. 2020	O-F-256565 = H1031, holotype	Spain	MN598116	n.a.	MN644506	n.a.	[22]
***H. phlebophoropsis*** X.C. Wang & W.Y. Zhuang, sp. nov.	HMAS 85654, holotype	China: Shanxi	**OQ597646**	**OQ600329**	n.a.	n.a.	This study
	HMAS 30757	China: Gansu	**OQ597647**	n.a.	n.a.	n.a.	This study
***H. plateata*** X.C. Wang & W.Y. Zhuang, sp. nov.	11229 = HMAS 290916	China: Tibet	**OQ597648**	**OQ600330**	**OQ586714**	**OQ597565**	This study
	HMAS 270642, holotype	China: Tibet	**OQ597649**	**OQ600331**	**OQ586715**	**OQ597566**	This study
	11595 = HMAS 290917	China: Yunnan	**OQ597650**	**OQ600332**	**OQ586716**	**OQ597567**	This study
	ZRL20201123 = HMAS 290918	China: Sichuan	**OQ597651**	**OQ600333**	**OQ586717**	n.a.	This study
*H. queletiana* Sacc. & Traverso 1910	C-F-45303 = H403, neotype	Denmark	KY784499	n.a.	KY773151	n.a.	[20]
***H. ravida*** X.C. Wang & W.Y. Zhuang, sp. nov.	10682 = HMAS 290919, holotype	China: Sichuan	**OQ597652**	**OQ600334**	**OQ586718**	**OQ597568**	This study
	HMAS 61920	China: Hebei	**OQ597653**	**OQ600335**	**OQ586719**	n.a.	This study
*H. rugosa* Q. Zhao & K.D. Hyde 2015	HKAS 75442, holotype	China: Yunnan	n.a.	JX462575	KR493511	n.a.	[47]
	HKAS 87587	China: Yunnan	n.a.	KR493478	n.a.	MG980690	[48]
	11596 = HMAS 290920	China: Yunnan	**OQ597654**	**OQ600336**	**OQ586720**	**OQ597569**	This study
	ChenZH 31346 = HMAS 290921	China: Yunnan	**OQ597655**	**OQ600337**	**OQ586721**	**OQ597570**	This study
	HMJAU 37659	China: Yunnan	**OQ597656**	**OQ600338**	**OQ586722**	**OQ597571**	This study
	11291 = HMAS 290922	China: Tibet	**OQ597657**	**OQ600339**	**OQ586723**	**OQ597572**	This study
	Wu 5321 = HMAS 290923	China: Guizhou	**OQ597658**	**OQ600340**	**OQ586724**	**OQ597573**	This study
	HMAS 72111	China: Guizhou	**OQ597659**	**OQ600341**	n.a.	n.a.	This study
	Zhang 7455 = HMAS 290924	China: Fujian	**OQ597660**	**OQ600342**	**OQ586725**	**OQ597574**	This study
	HMAS 270927	China: Guangdong	**OQ597661**	**OQ600343**	**OQ586726**	**OQ597575**	This study
	HMAS 270956	China: Guangdong	**OQ597662**	**OQ600344**	**OQ586727**	**OQ597576**	This study
	HMAS 270961	China: Guangdong	**OQ597663**	**OQ600345**	**OQ586728**	**OQ597577**	This study
	HMIGD 43032	China: Guangdong	**OQ597664**	**OQ600346**	**OQ586729**	**OQ597578**	This study
	HMIGD 70298	China: Guangdong	**OQ597665**	**OQ600347**	**OQ586730**	**OQ597579**	This study
	HMIGD 70454	China: Guangdong	**OQ597666**	**OQ600348**	**OQ586731**	**OQ597580**	This study
	HMIGD 70469	China: Guangdong	**OQ597667**	n.a.	n.a.	n.a.	This study
	8021 = HMAS 290925	China: Jilin	**OQ597668**	MG846999	MG847045	MG847091	This study, [32]
	8023 = HMAS 290926	China: Jilin	**OQ597669**	**OQ600349**	**OQ586732**	**OQ597581**	This study
	8024 = HMAS 290927	China: Jilin	**OQ597670**	**OQ600350**	**OQ586733**	**OQ597582**	This study
*H. semiobruta* Donadini & Berthet 1976	C-F-45467 = H307	Spain	KY784417	n.a.	KY773102	n.a.	[20]
*H. sublactea* Q. Zhao et al. 2016	HKAS 69753 = Zhao 1032, holotype	China: Yunnan	n.a.	KT894825	KT894832	n.a.	[31]
	C-F-45434 = H400	Papua New Guinea	KY784497	n.a.	n.a.	n.a.	[20]
	8022 = HMAS 290928	China: Jilin	**OQ597671**	**OQ600351**	**OQ586734**	**OQ597583**	This study
	CFSZ 11221	China: Inner Mongolia	**OQ597672**	**OQ600352**	**OQ586735**	**OQ597584**	This study
	CFSZ 2041	China: Inner Mongolia	**OQ597673**	**OQ600353**	n.a.	n.a.	This study
	CFSZ 4790	China: Inner Mongolia	**OQ597674**	n.a.	**OQ586736**	n.a.	This study
	HMAS 33914	China: Beijing	**OQ597675**	n.a.	n.a.	n.a.	This study
	HMAS 33915	China: Beijing	**OQ597676**	n.a.	n.a.	n.a.	This study
	HMAS 33916	China: Beijing	**OQ597677**	n.a.	n.a.	n.a.	This study
	HMAS 85702	China: Jilin	**OQ597678**	n.a.	n.a.	n.a.	This study
*H. sulcata* Afzel. 1783	O-68095 = H152, epitype	Norway	KY784284	n.a.	KY773001	KY772882	[20]
*H. terricola* Skrede & T. Schumach. 2020	O-F256562 = H2978, holotype	Spain	MN598197	n.a.	n.a.	n.a.	[22]
	HMAS 38355	China: Xinjiang	**OQ597679**	**OQ600354**	n.a.	n.a.	This study
***H. varia*** X.C. Wang & W.Y. Zhuang, sp. nov.	HMAS 131945	China: Yunnan	**OQ597680**	**OQ600355**	n.a.	n.a.	This study
	HMAS 186052	China: Yunnan	**OQ597681**	**OQ600356**	n.a.	n.a.	This study
	HMAS 270932, holotype	China: Guangdong	**OQ597682**	**OQ600357**	**OQ586737**	**OQ597585**	This study
	HMAS 270958	China: Guangdong	**OQ597683**	**OQ600358**	n.a.	**OQ597586**	This study
	Wu 345 = HMAS 290929	China: Guizhou	**OQ597684**	**OQ600359**	**OQ586738**	**OQ597587**	This study
	ZRL20150069 = HMAS 290930	China: Zhejiang	**OQ597685**	**OQ600360**	**OQ586739**	**OQ597588**	This study
	ZRL20191640 = HMAS 290931	China: Zhejiang	**OQ597686**	**OQ600361**	**OQ586740**	n.a.	This study
*H. vespertina* N.H. Nguyen & Vellinga 2013	UC 1999206	USA: California	n.a.	KC122847	KC122780	n.a.	[18]
	H102	USA: California	KY784245	n.a.	KY772963	n.a.	[20]
***H. vitrea*** X.C. Wang & W.Y. Zhuang, sp. nov.	ZhangZH02 = HMAS 290932, holotype	China: Jiangsu	**OQ597687**	**OQ600362**	**OQ586741**	n.a.	This study
***H. vulgata*** X.C. Wang & W.Y. Zhuang, sp. nov.	HMAS 53683, holotype	China: Hubei	**OQ597688**	**OQ600363**	**OQ586742**	n.a.	This study
	HMAS 85589	China: Jilin	**OQ597689**	**OQ600364**	n.a.	n.a.	This study
	HMIGD 25964	China: Jilin	**OQ597690**	**OQ600365**	**OQ586743**	**OQ597589**	This study
***H. yunnanensis*** X.C. Wang & W.Y. Zhuang, sp. nov.	11785 = HMAS 290933, holotype	China: Yunnan	**OQ597691**	**OQ600366**	**OQ586744**	**OQ597590**	This study
	11789 = HMAS 290934	China: Yunnan	**OQ597692**	**OQ600367**	**OQ586745**	**OQ597591**	This study
*Helvella* sp. 1	O-253390 = H213	Japan	KY784334	n.a.	KY773045	n.a.	[20]
*Helvella* sp. 2	O-253391 = H461	Japan	KY784543	n.a.	n.a.	n.a.	[20]
*Helvella* sp. 3	O-253393 = H466	Japan	KY784547	n.a.	n.a.	n.a.	[20]
*Helvella* sp. 4	H104	USA: Massachusetts	KY784247	n.a.	KY772964	KY772869	[20]
*Helvella* sp. 5	C-F-92119 = H377	USA: Michigan	KY784476	n.a.	KY773139	n.a.	[20]
*Helvella* sp. 6	UC 1999237 = MES286	USA: California	n.a.	KC122810	KC122773	n.a.	[18]
*Dissingia leucomelaena* (Pers.) K. Hansen & X.H. Wang 2019	KH.06.01 = H115	USA: Massachusetts	KY784253	n.a.	KC012682	KC109207	[20,49]
	HMAS 61356	Sweden	MK652202	MK592137	n.a.	n.a.	[21]
*D. oblongispora* (Harmaja) T. Schumach. & Skrede 2019	O-166316 = H132	Norway	KY784265	n.a.	KY772983	MK113836	[20,44]
	HMAS 75147	China: Sichuan	MK652205	MK592140	n.a.	MK652162	[21]

GenBank accession numbers in bold indicating newly generated sequences.

## 3. Results

### 3.1. Molecular Phylogenies

Sequences from 49 species of the *Helvella lacunosa* clade and two outgroup taxa were investigated (Table 1). The characteristics of each dataset, e.g., the number of sequences included, alignment length, and numbers of variable and informative sites, are given in Table 2.

**Figure 1:**
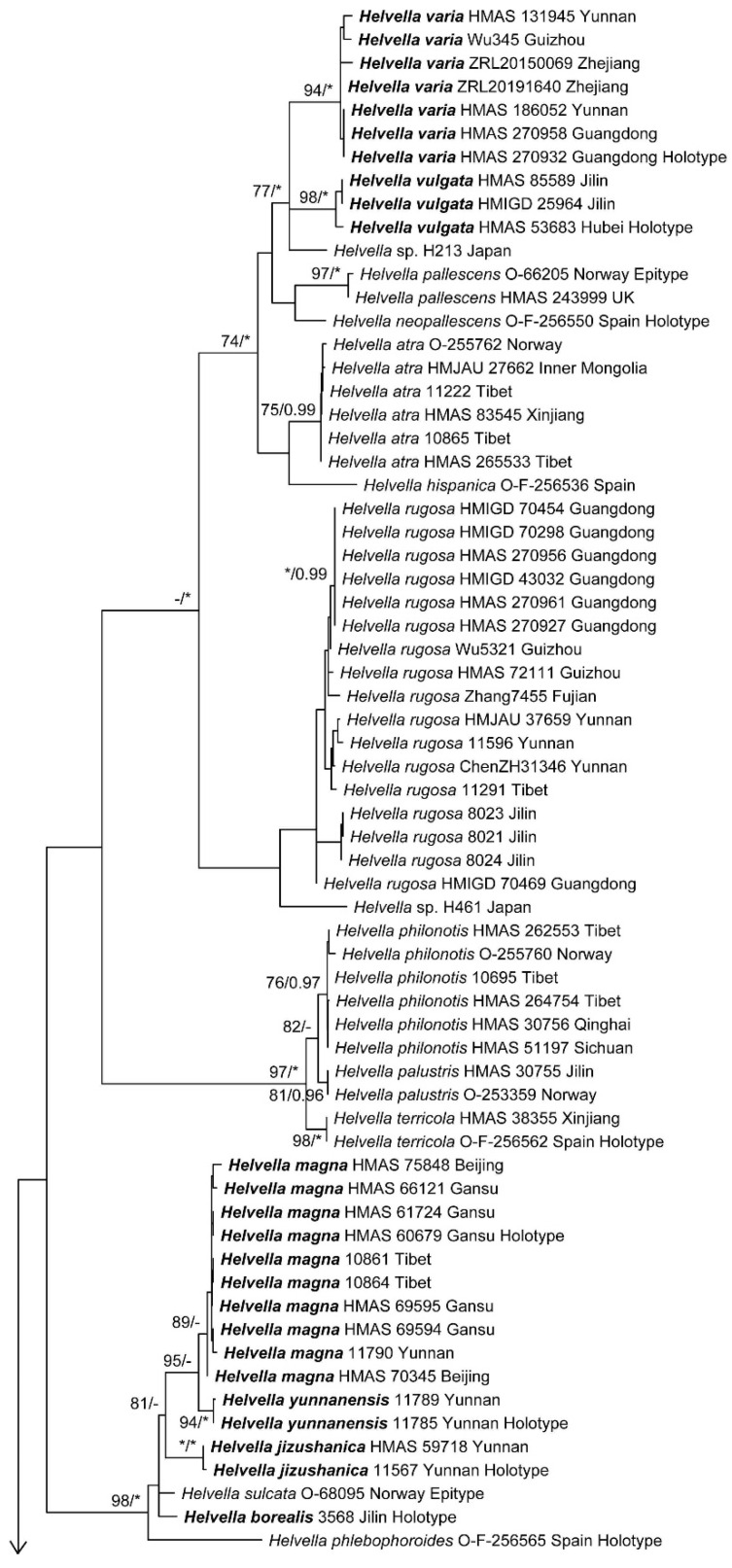

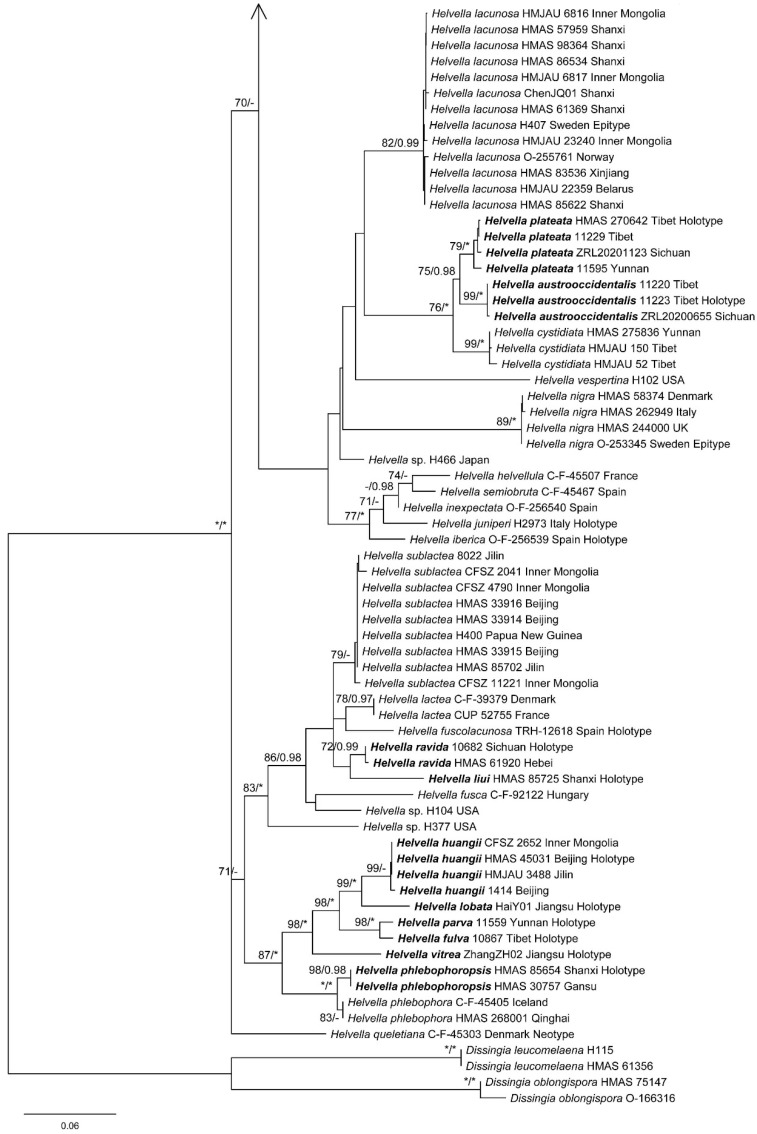
Maximum-likelihood phylogenetic tree of *Helvella lacunosa* clade inferred from combined Hsp90, ITS, LSU, and TEF1 dataset. Bootstrap values ≥ 70% (**left**) and posterior probability values ≥ 0.95 (**right**) are indicated at nodes. Asterisk denotes 100% bootstrap or 1.00 posterior probability.

**Figure 2:**
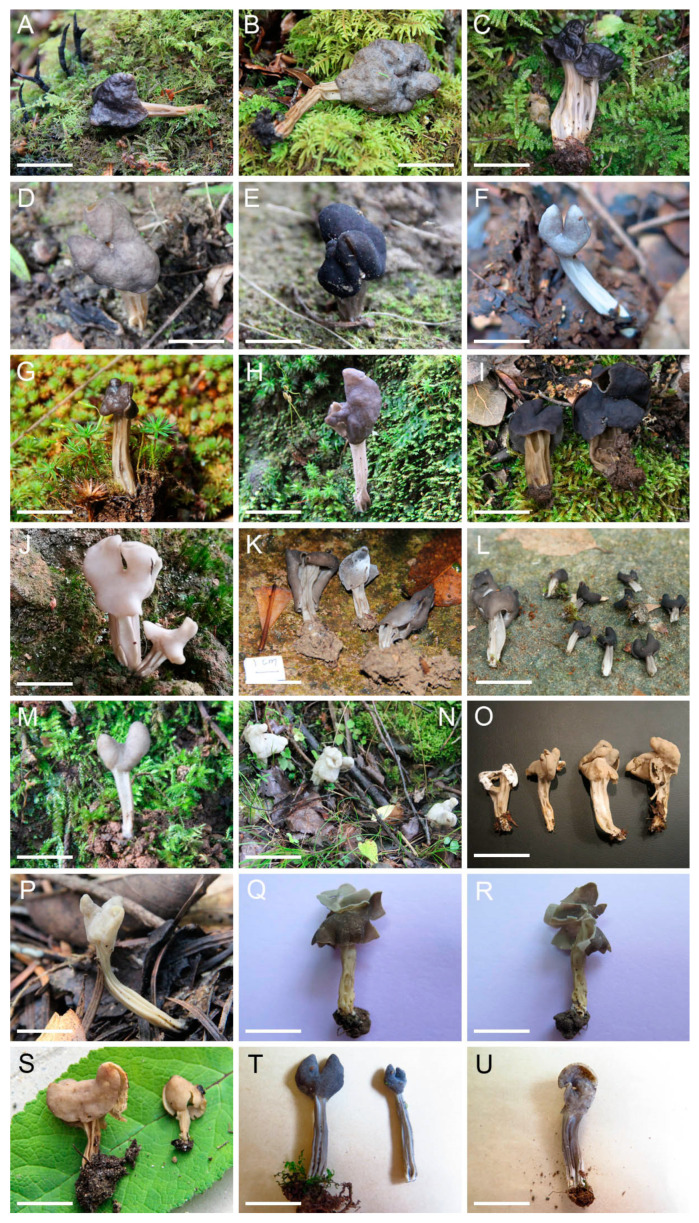
Photographs of fresh apothecia of *Helvella* species. (**A**–**C**) *H. austrooccidentalis*: (**A**) 11223 (holotype); (**B**) 11220; (**C**) ZRL20200655. (**D**–**F**) *H. magna*: (**D**,**E**) 10864; (**F**) 11790. (**G**–**I**) *H. plateata*: (**G**) 11229; (**H**) 11595; (**I**) ZRL20201123. (**J**–**L**) *H. varia*: (**J**) Wu345; (**K**) ZRL20150069; (**L**) ZRL20191640. (**M**) *H. parva* 11559 (holotype). (**N**,**O**) *H. ravida* 10682 (holotype). (**P**) *H. jizushanica* 11567 (holotype). (**Q**,**R**) *H. lobata* HaiY01 (holotype). (**S**) *H. fulva* 10867 (holotype). (**T**,**U**) *H. yunnanensis*: (**T**) 11785 (holotype); (**U**) 11789. Bars: (**B**,**N**) = 3 cm; (**A**) = 2.5 cm; (**H**,**K**,**L**,**O**,**P**,**S**,**T**,**U**) = 2 cm; (**I**) = 1.75 cm; (**C**,**F**,**G**,**J**) = 1.5 cm; (**Q**,**R**) = 1.2 cm; (**D**,**E**) = 1 cm; (**M**) = 0.75 cm.

**Figure 3:**
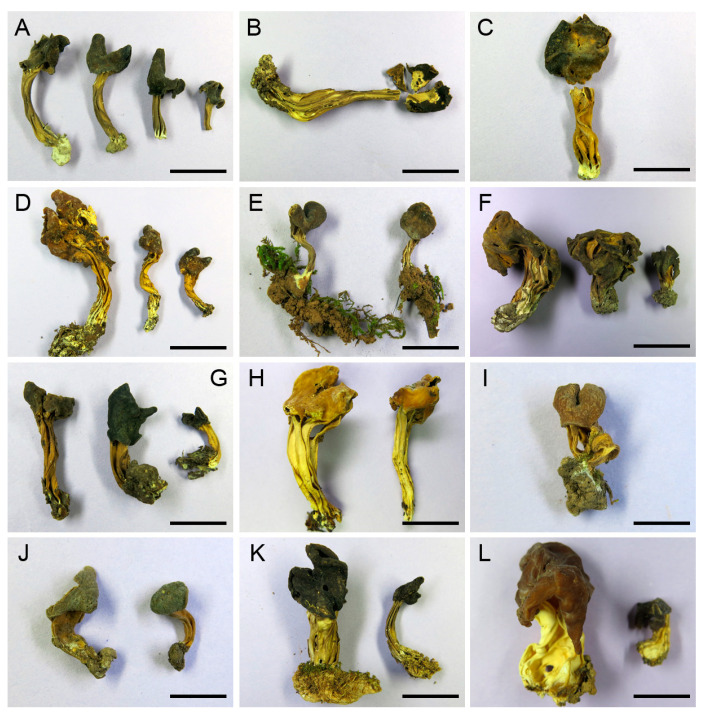
Photographs of dried apothecia of *Helvella* species. (**A**) *H. varia* HMAS 270932 (holotype). (**B**) *H. cystidiata* HMAS 275836. (**C**) *H. huangii* HMAS 45031 (holotype). (**D**) *H. borealis* 3568 (holotype). (**E**) *H. parva* 11559 (holotype). (**F**) *H. magna* HMAS 60679 (holotype). (**G**) *H. vulgata* HMAS 53683 (holotype). (**H**) *H. ravida* 10682 (holotype). (**I**) *H. liui* HMAS 85725 (holotype). (**J**) *H. phlebophoropsis* HMAS 85654 (holotype). (**K**) *H. plateata* HMAS 270642 (holotype). (**L**) *H. vitrea* ZhangZH02 (holotype). Bars: (**F**) = 3 cm; (**B**,**D**) = 1.5 cm; (**C**,**H**,**K**,**L**) = 1.2 cm; (**A**,**E**,**G**) = 1 cm; (**I**,**J**) = 0.7 cm.

**Figure 4:**
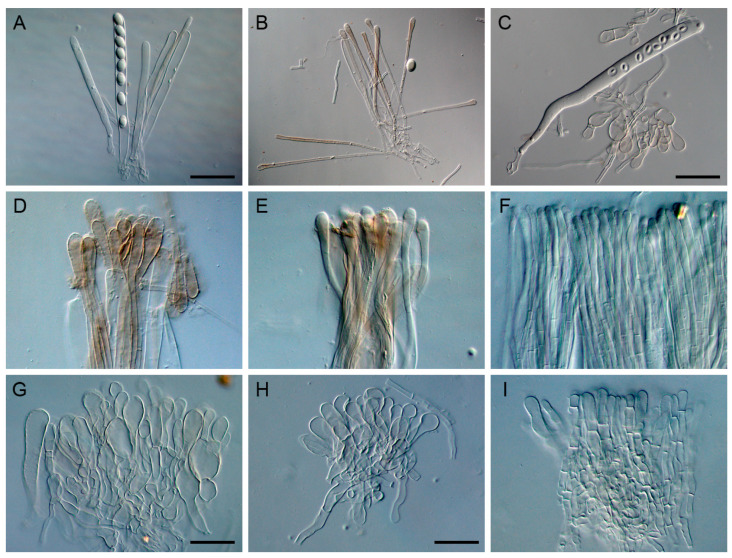
Microscopic characteristics of *Helvella* species. (**A**–**C**) Asci and paraphyses [(**A**) *H. parva* 11559 (holotype); (**B**,**C**) *H. varia* HMAS 131945]. (**D**–**F**) Paraphyses [(**D**) *H. austrooccidentalis* 11223 (holotype); (**E**) *H. magna* HMAS 60679 (holotype); (**F**) *H. liui* HMAS 85725 (holotype)]. (**G**–**I**) Outer cells of ectal excipulum [(**G**) *H. magna* 10861; (**H**) *H. fulva* 10867 (holotype); (**I**) *H. liui* HMAS 85725 (holotype)]. Bars: (**A**) = 60 µm, applied to (**B**); (**C**) = 45 µm; (**G**) = 40 µm; (**H**) = 30 µm, applied to (**D**–**F**,**I**).

**Figure 5:**
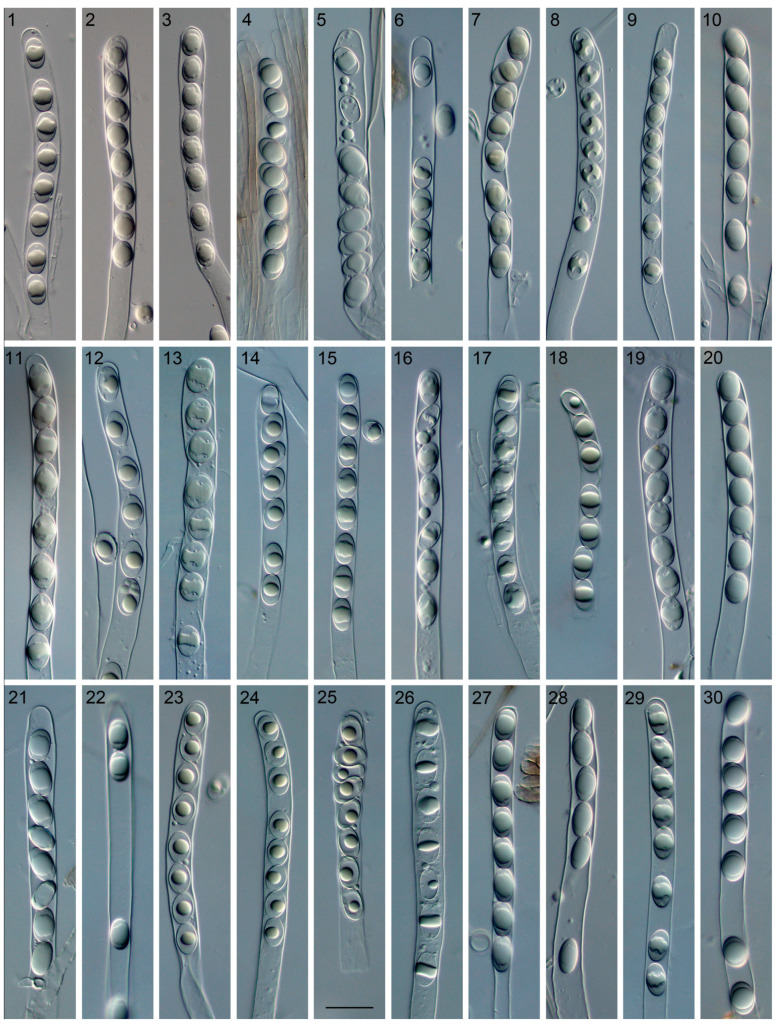
Ascospores in asci of *Helvella* species. From left to right in the top row: (**1**) *Helvella varia* HMAS 131945; (**2**) *H. varia* HMAS 270932 (holotype); (**3**) *H. varia* Wu 345; (**4**) *H. varia* ZRL20150069; (**5**) *H. cystidiata* HMAS 275836; (**6**) *H. cystidiata* HMJAU 150; (**7**) *H. huangii* HMAS 45031 (holotype); (**8**) *H. huangii* CFSZ 2652; (**9**) *H. borealis* 3568 (holotype); (**10**) *H. parva* 11559 (holotype); from left to right in the middle row: (**11**) *H. magna* 10861; (**12**) *H. magna* HMAS 60679 (holotype); (**13**) *H. magna* HMAS 70345; (**14**) *H. vulgata* HMAS 53683 (holotype); (**15**) *H. vulgata* HMIGD 25964; (**16**) *H. jizushanica* 11567 (holotype); (**17**) *H. ravida* 10682 (holotype); (**18**) *H. ravida* HMAS 61920; (**19**) *H. fulva* 10867 (holotype); (**20**) *H. lobata* HaiY01 (holotype); from left to right in the bottom row: (**21**) *H. austrooccidentalis* 11223 (holotype); (**22**) *H. austrooccidentalis* ZRL20200655; (**23**) *H. liui* HMAS 85725 (holotype); (**24**) *H. phlebophoropsis* HMAS 85654 (holotype); (**25**) *H. phlebophoropsis* HMAS 30757; (**26**) *H. plateata* HMAS 270642 (holotype); (**27**) *H. plateata* 11595; (**28**) *H. plateata* ZRL20201123; (**29**) *H. yunnanensis* 11785 (holotype); (**30**) *H. vitrea* ZhangZH02 (holotype). Bar = 30 µm, applied to all the figures.

The four-locus dataset contained 135 sequences in an alignment with a length of 2652 base pairs (bp). A transversion model with invariable sites and gamma distribution (TVM+I+G) was selected by means of the Akaike information criterion as the best fit for Bayesian inference analysis. The phylogeny of this clade was reconstructed (Figure 1). A total of 46 *Helvella* lineages were recognized, and 25 of them consisted of Chinese materials. Nine lineages stood for the following known species: *H. atra*, *H. cystidiata*, *H. lacunosa*, *H. palustris*, *H. philonotis*, *H. phlebophora*, *H. rugosa*, *H. sublactea*, and *H. terricola*. The remaining 16 species represented undescribed taxa. The macro- and microscopic morphological characters of the undescribed species were given in Figure 2, Figure 3, Figure 4 and Figure 5.

The phylogenies inferred from the individual gene datasets are shown in Figures S1–S4. Compared with the multi-locus phylogeny (Figure 1), three additional lineages were revealed in the ITS and LSU trees, representing *H. dryophila*, *H. jocatoi*, and *Helvella* sp. UC1999237. They clustered with *H. vespertina,* forming a subclade with high statistic supports (MLBP = 99% in Figure S2, MLBP = 98% in Figure S3). Additionally, they were all from North America. Hsp90 phylogeny showed less robust clustering support than the multi-locus or ITS ones (Figure 1 and Figure S1). The TEF tree shared a similar topology with the four-locus and ITS phylogenies (Figure S4).

### 3.2. Taxonomy

#### 3.2.1. New Species

***Helvella austrooccidentalis*** X.C. Wang & W.Y. Zhuang, sp. nov. Figure 2A–C, Figure 4D and Figure 5(21–22)

Fungal Names: FN571317

Etymology: The specific epithet refers to geographic distribution of the species in southwestern China.

Typification: China, Tibet, Nyingchi City, Bayi District, Lulang Town, 29°46′3″ N, 94°44′3″ E, on rotten trunk, 23 September 2016, Xin-Cun Wang et al. 11223, HMAS 290903, holotype.

Apothecia saddle-shaped or lobed, stipitate, 3–4 cm diam. and 5.5–10 cm high when fresh, 1.6–2.5 cm diam. and 4.5–7 cm high when dry; margin revolute or attached to the stipe; hymenium surface undulate-rugose, greyish brown, dark brown to black when fresh, dark brown to black when dry; receptacle surface light brown to dark brown when dry, glabrous; stipe surface ribbed or lacunose, dirty white to yellow brown when fresh, yellow brown to light brown when dry, 3.5–5 × 0.2–0.7 cm when dry. Ectal excipulum of textura angularis, cells hyaline to yellow brown, outer cells 12–46.5 × 6–20 µm. Medullary excipulum of textura intricata, hyphae hyaline. Asci subcylindrical, tapering at base, eight-spored, 260–400 × 13–21 µm. Paraphyses filiform to clavate, septate, hyaline to yellow brown, 5–13 µm wide at apex and 2.5–4 µm below. Ascospores narrow ellipsoidal to ellipsoidal, hyaline, smooth, uniguttulate, 16–22 × 10.5–14.5 µm, Q = 1.5–1.65.

Additional specimens examined: China, Sichuan Province, Garzê Tibetan Autonomous Prefecture, Yajiang County, Gexigou National Nature Reserve, Hekou Town, Xiadu Village, 29°57′25″ N, 100°57′35″ E, Alt. 3241.56 m, on ground of mixed forest, Rui-Lin Zhao et al. ZRL20200655, HMAS 290904. Tibet, Nyingchi City, Bayi District, Lulang Town, 29°46′3″ N, 94°44′3″ E, on rotten trunk, 23 September 2016, Xin-Cun Wang et al. 11220, HMAS 290902.

Notes: This species was a sister of *H. cystidiata* and *H. plateata* (Figure 1). It differs from *H. cystidiata* in 3 bp for Hsp 90, 121 bp for ITS (112 bp in ITS1, 1 bp in 5.8S, and 8 bp in ITS2), 5 bp for LSU, and 4 bp for TEF; it differs from *H. plateata* in 1 bp for Hsp90, 105 bp for ITS (93 bp in ITS1, 1 bp in 5.8S, and 11 bp in ITS2), 4 bp for LSU, and 1 bp for TEF. Morphologically, it differs from *H. cystidiata* in yellow brown paraphysis apex and differs from *H. plateata* in lighter paraphysis color and broader ascospores (Table 3).

***Helvella borealis*** X.C. Wang & W.Y. Zhuang, sp. nov. Figure 3D and Figure 5(9)

Fungal Names: FN571318

Etymology: The specific epithet refers to the geographic distribution of the species.

Typification: China, Jilin Province, Yanbian Korean Autonomous Prefecture, Dunhua City, Huangnihe National Nature Reserve, Donggou, 43°55′6″ N, 128°18′57″ E, Alt. 350 m, on rotten wood, 17 August 2000, Wen-Ying Zhuang and Yan-Hui Zhang 3568, HMAS 290905, holotype.

Apothecia saddle-shaped, stipitate, 0.4–1.5 cm diam. and 1–4 cm high when dry; margin revolute or attached to the stipe; hymenium surface undulate-rugose, dirty white to greyish white when fresh, yellow brown, red brown to dark brown when dry; receptacle surface buff when dry, glabrous; stipe surface sulcate, whitish when fresh, buff to yellow brown when dry, 2.3–5.5 cm when fresh, 0.7–3 × 0.2–0.5 cm when dry. Ectal excipulum of textura angularis, cells hyaline to light brown, outer cells 17–73 × 6.5–37 µm. Medullary excipulum of textura intricata, hyphae hyaline. Asci subcylindrical, tapering at base, eight-spored, 240–280 × 12–14.5 µm. Paraphyses filiform, septate, hyaline, 6.5–10.5 µm wide at apex and 3.5–5 µm below. Ascospores ellipsoidal, hyaline, smooth, uniguttulate, 14.5–16 × 9–10.5 µm, Q = 1.55.

Notes: *Helvella sulcata* was treated as a synonym of *H. lacunosa* by Dissing [3] and some subsequent researchers [5,50] but accepted as a separate species by Weber [4]. *Helvella borealis* has the closest relationship with *H. sulcata* (Figure 1), but differs in 1 bp for Hsp90, 3 bp for LSU, and 5 bp for TEF. Morphologically, *H. borealis* differs from *H. sulcata* in white or brown but not black hymenium, shorter asci (240–280 vs. 290–320 µm), broader paraphyses apex (6.5–10.5 vs. 5–6.5 µm), and narrower ascospores (9–10.5 vs. 10.5–13.2 µm) [22].

***Helvella fulva*** X.C. Wang & W.Y. Zhuang, sp. Nov. Figure 2S, Figure 4H and Figure 5(19)

Fungal Names: FN571319

Etymology: The specific epithet refers to the hymenium color of the species.

Typification: China, Tibet, Nyingchi City, Mainling County, Lilong Town, Lilonggou, 29°2′23″ N, 93°53′41″ E, on soil, 14 September 2016, Xin-Cun Wang et al. 10867, HMAS 290906, holotype.

Apothecia saddle-shaped or lobed, stipitate, 1.6–3 cm diam. and 2.3–3.8 cm high when fresh; margin revolute or attached to the stipe; hymenium surface undulate-rugose, light brown when fresh, reddish brown to dark brown when dry; receptacle surface yellow brown when dry, glabrous; stipe surface sulcate, yellow brown to light brown when dry, 1–1.5 × 0.2–0.4 cm when dry. Ectal excipulum of textura angularis, cells hyaline, outer cells 24–46.5 × 8–18.5 µm. Medullary excipulum of textura intricata, hyphae hyaline. Asci subcylindrical, tapering at base, eight-spored, 280–346.5 × 13–21 µm. Paraphyses filiform to clavate, septate, hyaline to yellow brown, 5–9 µm wide at apex and 2.5–4 µm below. Ascospores ellipsoidal, hyaline, smooth, uniguttulate, 17–20 × 10.5–12.5 µm, Q = 1.6.

Notes: This species is a sister of *H. parva* (Figure 1), but differs in 1 bp in Hsp90, 14 bp for ITS (10 bp in ITS1 and 4 bp in ITS2), 3 bp for LSU, and 6 bp for TEF. Morphologically, it has longer asci and shorter outer ectal excipular cells than those of the latter (Table 3).

***Helvella huangii*** X.C. Wang & W.Y. Zhuang, sp. nov. Figure 3C and Figure 5(7–8)

Fungal Names: FN571321

Etymology: The specific epithet is named after the distinguished Chinese mycologist Nian-Lai Huang (1939.11–2022.09, Sanming Mycological Institute).

Typification: China, Beijing City, Mentougou District, Tanzhe Temple, 39°54′23″ N, 116°1′54″ E, on soil, 23 August 1982, Ru-Yong Zheng and Wen-Ying Zhuang ZC 8, HMAS 45031, holotype.

Apothecia saddle-shaped, stipitate, 0.7–1.5 cm diam. and 2–3.3 cm high when dry; margin revolute or attached to the stipe; hymenium surface undulate-rugose, yellow brown, red brown, light brown, dark brown or black when dry; receptacle surface yellow brown, light brown to dark brown when dry, glabrous; stipe surface sulcate, buff, yellow brown to light brown when dry, 1.3–2.4 × 0.2–0.6 cm when dry. Ectal excipulum of textura angularis, cells hyaline to light brown, outer cells 20–40 × 8–18.5 µm. Medullary excipulum of textura intricata, hyphae hyaline. Asci subcylindrical, tapering at base, eight-spored, 213.5–320 × 13.5–18.5 µm. Paraphyses filiform, septate, hyaline to yellow brown, 4–8 µm wide at apex and 2.5–5.5 µm below. Ascospores ellipsoidal, hyaline, smooth, uniguttulate, 14.5–20 × 10.5–13 µm, Q = 1.5.

Additional specimens examined: China, Beijing City, Mentougou District, Tanzhe Temple, 39°54′23″ N, 116°1′54″ E, Alt. 400 m, on ground, 17 September 1996, Zheng Wang and Wen-Ying Zhuang 1414, HMAS 290907. Inner Mongolia Autonomous Region, Chifeng City, Bairin Left Banner, Yezhugou Village, 43°43′55″ N, 119°21′38″ E, 16 August 2005, Tie-Zhi Liu and Yu-Jun Gao, CFSZ 2652. Jilin Province, Changchun City, Jingyuetan National Forest Park, 43°46′41″ N, 125°28′25″ E, on the ground of broad-leaved forest, 15 August 2004, Jian-Rui Wang, HMJAU 3488.

Notes: The species is a sister of *H. lobata* (Figure 1) but differs from the latter in 1 bp for Hsp90, 49 bp for ITS (34 bp in ITS1 and 15 bp in ITS2), and 6 bp for LSU. Morphologically, it has narrower ascospores than that of the latter (Table 3).

***Helvella jizushanica*** X.C. Wang & W.Y. Zhuang, sp. nov. Figure 2P and Figure 5(16)

Fungal Names: FN571322

Etymology: The specific epithet refers to the type locality of the fungus.

Typification: China, Yunnan Province, Dali Bai Autonomous Prefecture, Binchuan County, Jizu Mountain, 25°57′27″ N, 100°23′34″ E, on soil, 20 September 2017, Xin-Cun Wang et al. 11567, HMAS 290908, holotype.

Apothecia saddle-shaped, stipitate, 1.5 cm diam. and 5 cm high when fresh, 0.5–0.8 cm diam. and 1.5–3.5 cm high when dry; margin revolute or attached to the stipe; hymenium surface undulate-rugose, yellow brown to greyish brown when fresh, light brown or dark brown when dry; receptacle surface white, buff or yellow brown when dry, glabrous; stipe surface sulcate, buff to light brown when dry, 1–3 × 0.15–0.35 cm when dry. Ectal excipulum of textura angularis, cells hyaline, outer cells 17–46.5 × 10.5–29 µm. Medullary excipulum of textura intricata, hyphae hyaline. Asci subcylindrical, tapering at base, eight-spored, 246.5–320 × 13–18.5 µm. Paraphyses filiform to clavate, septate, hyaline to yellow brown, 6–8.5 µm wide at apex and 2.5 µm below. Ascospores ellipsoidal, hyaline, smooth, uniguttulate, 16–21 × 10.5–12.5 µm, Q = 1.6.

Additional specimen examined: China, Yunnan Province, Dali Bai Autonomous Prefecture, Binchuan County, Jizu Mountain, on soil, 8 August 1989, Yu-Chen Zong and Yu Li 176, HMAS 59718.

Notes: This species has close relationships with *H. magna* and *H. yunnanensis* (Figure 1). It differs from *H. magna* in 2 bp for Hsp90, 31 bp for ITS (16 bp in ITS1 and 15 bp in ITS2), 11 bp for LSU, and 3 bp for TEF; and from *H. yunnanensis* in 1 bp for Hsp90, 32 bp for ITS (15 bp in ITS1 and 17 bp in ITS2), 12 bp for LSU, and 2 bp for TEF. Morphologically, the ascomata of this species have a yellower tint than those of its allies.

***Helvella liui*** X.C. Wang & W.Y. Zhuang, sp. nov. Figure 3I, Figure 4F,I and Figure 5(23)

Fungal Names: FN571323

Etymology: The specific epithet is named after the distinguished Chinese mycologist Bo Liu (1927.03–2017.07, Shanxi University).

Typification: China, Shanxi Province, Taiyue Mountain National Forest Park, on the ground of forest, 12 August 1985, Fu Du, MHSU 455 = HMAS 85725, holotype.

Apothecia saddle-shaped, stipitate, 0.7 cm diam. and 1 cm high when dry; margin revolute or attached to the stipe; hymenium surface undulate-rugose, orange brown when dry; receptacle surface yellow brown when dry, glabrous; stipe surface sulcate, yellow brown when dry, 0.5 × 0.15 cm when dry. Ectal excipulum of textura angularis, cells hyaline to yellow brown, outer cells 20–33 × 5–10.5 µm. Medullary excipulum of textura intricata, hyphae hyaline. Asci subcylindrical, tapering at base, eight-spored, 266.5–333 × 16–18.5 µm. Paraphyses filiform, septate, hyaline, 5–6.5 µm wide at apex and 3–4 µm below. Ascospores ellipsoidal, hyaline, smooth, uniguttulate, 17–20 × 11–13 µm, Q = 1.5.

Notes: This species is sister of *H. ravida* (Figure 1) but differs from the latter in 4 bp for Hsp90 and 16 bp for ITS2. Although both of them have hyaline paraphyses, this species has narrower paraphyses and smaller outer ectal excipular cells than the latter (Table 3).

***Helvella lobata*** X.C. Wang & W.Y. Zhuang, sp. nov. Figure 2Q–R and Figure 5(20)

Fungal Names: FN571324

Etymology: The specific epithet refers to the lobed apothecia of this species.

Typification: China, Jiangsu Province, Nanjing City, Xuanwu District, Zijin Mountain, 32°4′17″ N, 118°50′57″ E, on soil, 30 May 2021, Ying Hai HaiY01, HMAS 290910, holotype.

Apothecia lobed, stipitate, 0.8 cm diam. and 2.8 cm high when dry; margin revolute or attached to the stipe; hymenium surface undulate-rugose, brown when fresh, dark brown to black when dry; receptacle surface greyish when fresh, whitish or blackish when dry, glabrous; stipe surface sulcate, whitish when fresh, yellow brown to light brown when dry, 2 × 0.2–0.3 cm when dry. Ectal excipulum of textura angularis, cells hyaline to yellow brown, outer cells 24–46.5 × 8–18.5 µm. Medullary excipulum of textura intricata, hyphae hyaline. Asci subcylindrical, tapering at base, eight-spored, 220–320 × 12–20 µm. Paraphyses filiform, septate, hyaline to yellow brown, 5–6.5 µm wide at apex and 2.5–4 µm below. Ascospores ellipsoidal, hyaline, smooth, uniguttulate, 16–20 × 12–14.5 µm, Q = 1.35.

Notes: This species is a sister of *H. huangii* (Figure 1). Their molecular and morphological distinctions were previously indicated in the notes of the latter.

***Helvella magna*** X.C. Wang & W.Y. Zhuang, sp. nov. Figure 2D–F, Figure 3F, Figure 4E,G and Figure 5(11–13)

Fungal Names: FN571326

Etymology: The specific epithet refers to the presence of large-sized ascomata of the species.

Typification: China, Gansu Province, Gannan Tibetan Autonomous Prefecture, Têwo County, Lazikou Town, Lazikou National Forest Park, 34°7′3″ N, 103°53′51″ E, Alt. 2050 m, on soil, 9 September 1992, Wen-Ying Zhuang and Xiao-Lan Mao 984, HMAS 60679, holotype.

Apothecia saddle-shaped or lobed and sometimes capitate, stipitate, 1.2–9 cm diam. and 3–8 cm high when fresh, 0.5–5 cm diam. and 1.5–6 cm high when dry; margin revolute or attached to the stipe; hymenium surface undulate-rugose, dark grey to black when fresh, greyish white, dirty grey, reddish brown, light brown, dark brown to black when dry; receptacle surface buff, yellow brown or blackish when dry, glabrous; stipe surface sulcate, dirty white, buff, yellow brown, reddish brown, light brown or dark brown when dry, 1–4 × 0.15–2.4 cm when dry. Ectal excipulum of textura angularis, cells hyaline to yellow brown, outer cells 20–88 × 8–30.5 µm. Medullary excipulum of textura intricata, hyphae hyaline. Asci subcylindrical, tapering at base, eight-spored, 280–415 × 13–20 µm. Paraphyses filiform to clavate, septate, hyaline to yellow brown, 3–13 µm wide at apex and 2.5–6 µm below. Ascospores ellipsoidal to broad ellipsoidal, hyaline, smooth, uniguttulate, 15–22.5 × 10.5–16 µm, Q = 1.35–1.65.

Additional specimens examined: China, Beijing City, Mentougou District, Qingshui Town, Baihua Mountain National Nature Reserve, 39°49′39″ N, 115°35′31″ E, Alt. 1400 m, on soil, Wen-Ying Zhuang and Zheng Wang 1215, HMAS 70345; *ibid.*, Dongling Mountain, 40°1′1″ N, 115°29′25″ E, Alt. 1100 m, on soil, 20 August 1998, Zheng Wang 282, HMAS 75848. Gansu Province, Gannan Tibetan Autonomous Prefecture, Têwo County, Dianga Town, Dalonggou, Alt. 2600 m, on soil, 10 September 1992, Wen-Ying Zhuang and Xiao-Qing Zhang 1005, HMAS 69595; *ibid.*, Lazikou Town, Lazikou National Forest Park, 34°7′3″ N, 103°53′51″ E, Alt. 2000 m, on the ground of mixed forest, 9 September 1992, Mao-Lin Tian M6470, HMAS 66121; *ibid.*, Zhouqu County, Wuping Town, Shatan Forest Farm, 33°41′48″ N, 104°10′14″ E, Alt. 2400 m, on soil, 3 September 1992, Wen-Ying Zhuang and Xiao-Qing Zhang 937, HMAS 69594; Longnan City, Huixian County, on soil, September 1992, Mao-Lin Tian M6510, HMAS 61724. Tibet, Nyingchi City, Mainling County, Wolong Town, 29°7′45″ N, 93°41′59″ E, on soil, 14 September 2016, Xin-Cun Wang et al. 10861, HMAS 290911; *ibid.*, 10864, HMAS 290912. Yunnan Province, Kunming City, Panlong District, Yeya Lake, 25°7′22″ N, 102°51′36″ E, on soil, 25 September 2017, Huan-Di Zheng et al. 11790, HMAS 290913.

Notes: This fungus is relatively common in China. Morphological variations were observed within species: some collections (HMAS 60679, 61724, and 66121) have large, rugose, and capitate apothecia and coarse and sulcate stipes, whereas others possess smaller non-capitate apothecia with somewhat slenderer stipes. It has close relationships with *H. jizushanica* and *H. yunnanensis* (Figure 1) but differs from *H. jizushanica* in 2 bp for Hsp90, 31 bp for ITS (16 bp in ITS1 and 15 bp in ITS2), 11 bp for LSU, and 3 bp for TEF; it differs from *H. yunnanensis* in 1 bp for Hsp90, 12 bp for ITS (7 bp in ITS1 and 5 bp in ITS2), 3 bp for LSU, and 1 bp for TEF. Collections having large capitate apothecia are easily distinguished from related species.

***Helvella parva*** X.C. Wang & W.Y. Zhuang, sp. nov. Figure 2M, Figure 3E, Figure 4A and Figure 5(10)

Fungal Names: FN571327

Etymology: The specific epithet refers to the small-sized ascomata of the species.

Typification: China, Yunnan Province, Dali Bai Autonomous Prefecture, Binchuan County, Jizu Mountain, 25°57′27″ N, 100°23′34″ E, on soil, 20 September 2017, Xin-Cun Wang et al. 11559, HMAS 290914, holotype.

Apothecia saddle-shaped or discoid, stipitate, 0.8–1 cm diam. and 2 cm high when fresh, 0.5 cm diam. and 1.1–1.2 cm high when dry; margin revolute or attached to the stipe; hymenium surface undulate-rugose, greyish when fresh, light brown to dark brown when dry; receptacle surface buff when dry, glabrous; stipe surface sulcate, greyish white when fresh, buff to light brown when dry, 0.8 × 0.1–0.2 cm when dry. Ectal excipulum of textura angularis, cells hyaline, outer cells 20–53 × 6.5–20 µm. Medullary excipulum of textura intricata, hyphae hyaline. Asci subcylindrical, tapering at base, eight-spored, 226.5–266.5 × 13–17 µm. Paraphyses filiform, septate, hyaline to yellow brown, 6.5 µm wide at apex and 3.5–4 µm below. Ascospores ellipsoidal, hyaline, smooth, uniguttulate, 17–18.5 × 10.5–12 µm, Q = 1.6.

Notes: This species was a sister to *H. fulva* (Figure 1). Their molecular and morphological differences were previously indicated in the notes of the latter.

***Helvella phlebophoropsis*** X.C. Wang & W.Y. Zhuang, sp. nov. Figure 3J and Figure 5(24–25)

Fungal Names: FN571328

Etymology: The specific epithet refers to its morphological similarity and phylogenetically close relationship with *H. phlebophora*.

Typification: China, Shanxi Province, Taiyue Mountain National Forest Park, 12 August 1985 Fu Du, MHSU 453 = HMAS 85654, holotype.

Apothecia discoid, stipitate, 0.5–1.2 cm diam. and 0.7–1.5 cm high when dry; margin revolute, attached to the stipe or not; hymenium surface undulate-rugose, dark brown to black when dry; receptacle surface dark brown to black when dry, glabrous; stipe surface ribbed, yellow brown to light brown when dry, 0.4–1.1 × 0.15–0.4 cm when dry. Ectal excipulum of textura angularis, cells hyaline to yellow brown, outer cells 24–42.5 × 5–12 µm. Medullary excipulum of textura intricata, hyphae hyaline. Asci subcylindrical, tapering at base, eight-spored, 240–280 × 13–20 µm. Paraphyses filiform, septate, hyaline to yellow brown, 4–8 µm wide at apex and 2.5–4 µm below. Ascospores ellipsoidal, hyaline, smooth, uniguttulate, 14.5–21 × 10.5–13 µm, Q = 1.5–1.6.

Additional specimen examined: China, Gansu Province, Tianshui City, Maiji District, Dongcha Town, Baiyanglin, 34°20′26″ N, 106°31′0″ E, on soil, 4 August 1958, Yu-Chuan Yang, 469, HMAS 30757.

Notes: It is a sister of *H. phlebophora* (Figure 1) but differs in 2 bp for Hsp90 and 2 bp for ITS2. Morphologically, it has wider asci (13–20 µm wide vs. 12–14 µm [3] or 12–15 µm [4] wide) and larger ascospores (14.5–21 × 10.5–13 µm vs. 15–16 × 9–10 µm [3] or 16–17.5 × 11–12 µm [4]) than the latter.

***Helvella plateata*** X.C. Wang & W.Y. Zhuang, sp. nov. Figure 2G–I, Figure 3K and Figure 5(26–28)

Fungal Names: FN571329

Etymology: The specific epithet refers to its location in the Qinghai–Tibet Plateau.

Typification: China, Tibet, Nyingchi City, Bayi District, Lulang Town, 29°45′57″ N, 94°44′28″ E, Alt. 3325 m, on rotten wood, 11 August 2013, Tie-Zheng Wei et al. 3655, HMAS 270642, holotype.

Apothecia saddle-shaped or lobed, stipitate, 0.8–2.7 cm diam. and 2.7–5 cm high when fresh, 0.4–1.6 cm diam. and 1.7–3.6 cm high when dry; margin revolute or attached to the stipe; hymenium surface undulate-rugose, light brown to black when fresh, dark brown to black when dry; receptacle surface light brown when dry, glabrous; stipe surface sulcate, light brown or dark brown when dry, 1.4–2.7 × 0.15–0.8 cm when dry. Ectal excipulum of textura angularis, cells hyaline to light brown, outer cells 18.5–60 × 6.5–24 µm. Medullary excipulum of textura intricata, hyphae hyaline. Asci subcylindrical, tapering at base, eight-spored, 206.5–426.5 × 13–20 µm. Paraphyses filiform to clavate, septate, hyaline to light brown, 4–10.5 µm wide at apex and 2.5–5 µm below. Ascospores narrow ellipsoidal, hyaline, smooth, uniguttulate, 16–23 × 9–13 µm, Q = 1.7–1.75.

Additional specimens examined: China, Sichuan Province, Garzê Tibetan Autonomous Prefecture, Batang County, Zhubalong Nature Reserve, 29°38′20″ N, 99°7′57″ E, Alt. 4274 m, on soil, 18 August 2020, Xin-Yu Zhu and Ming-Zhe Zhang ZRL20201123, HMAS 290918. Tibet, Nyingchi City, Bayi District, Lulang Town, 29°46′3″ N, 94°44′3″ E, on soil, 23 September 2016, Xin-Cun Wang et al. 11229, HMAS 290916. Yunnan Province, Dali Bai Autonomous Prefecture, Binchuan County, Jizu Mountain, 25°57′46″ N, 100°22′41″ E, on soil, 21 September 2017, Xin-Cun Wang et al. 11595, HMAS 290917.

Notes: This species is a sister of *H. austrooccidentalis* and *H. cystidiata* (Figure 1). It differs from *H. cystidiata* in 2 bp for Hsp90, 61 bp for ITS (47 bp in ITS1, 2 bp in 5.8S, and 12 bp in ITS2), 2 bp for LSU, and 3 bp for TEF. The molecular distinction between *H. plateata* and *H. austrooccidentalis* was discussed in the notes of the latter. This species possesses very narrow ascospores compared with the others (Table 3).

***Helvella ravida*** X.C. Wang & W.Y. Zhuang, sp. nov. Figure 2N,O, Figure 3H and Figure 5(17–18)

Fungal Names: FN571330

Etymology: The specific epithet refers to color of the apothecia of this species.

Typification: China, Sichuan Province, Ngawa Tibetan and Qiang Autonomous Prefecture, Li County, Miyaluo Town, Jiabigou, 31°37′32″ N, 102°50′23″ E, Alt. 2850 m, on the ground of *Picea* sp. forest, 10 August 2016, Xin-Cun Wang 10682, HMAS 290919, holotype.

Apothecia saddle-shaped or lobed, stipitate, 1.2–2.1 cm diam. and 2.2–4.5 cm high when dry; margin revolute or attached to the stipe; hymenium surface undulate-rugose, greyish white to light grey when fresh, yellow brown, orange brown or dark brown when dry; receptacle surface yellow brown, light brown or dark brown when dry, glabrous; stipe sulcate, buff, yellow brown to light brown when dry, 1.5–3.5 × 0.2–0.8 cm when dry. Ectal excipulum of textura angularis, cells hyaline to yellow brown, outer cells 17–57 × 8–25 µm. Medullary excipulum of textura intricata, hyphae hyaline. Asci subcylindrical, tapering at base, eight-spored, 240–333 × 14.5–24 µm. Paraphyses filiform to clavate, septate, hyaline to yellow brown, 8–13 µm wide at apex and 4–4.5 µm below. Ascospores ellipsoidal, hyaline, smooth, uniguttulate, 16–20 × 10.5–13 µm, Q = 1.5.

Additional specimen examined: China, Hebei Province, Zhangjiakou City, Xiaowutai Mountain National Nature Reserve, Xitai, 39°54′49″ N, 114°58′4″ E, on soil, 29 August 1990, Hua-An Wen and Bin Li 273, HMAS 61920.

Notes: This species is a sister of *H. liui* (Figure 1). Their molecular and morphological distinctions were indicated in the notes of the latter.

***Helvella varia*** X.C. Wang & W.Y. Zhuang, sp. nov. Figure 2J–L, Figure 3A, Figure 4B,C and Figure 5(1–4)

Fungal Names: FN571331

Etymology: The specific epithet refers to the varied color of the apothecia of this species.

Typification: China, Guangdong Province, Shaoguan City, Ruyuan Yao Autonomous County, Nanling National Nature Reserve, Xiaohuangshan, 24°53′47″ N, 113°1′8″ E, Alt. 1350 m, on the ground of broad-leaved forest, 15 June 2014, Tie-Zheng Wei 3914, HMAS 270932, holotype.

Apothecia saddle-shaped or irregularly lobed, stipitate, 0.3–2.3 cm diam. and 0.8–6 cm high when dry; margin revolute or attached to the stipe; hymenium surface undulate-rugose, white to blackish when fresh, yellow brown to black when dry; receptacle surface buff to dark brown when dry, glabrous; stipe surface sulcate, buff to light brown when dry, 0.5–4.5 × 0.15–0.65 cm when dry. Ectal excipulum of textura angularis, cells hyaline to light brown, outer cells 14.5–46.5 × 9–21.5 µm. Medullary excipulum of textura intricata, hyphae hyaline. Asci subcylindrical, tapering at base, eight-spored, 195–305 × 13.5–22.5 µm. Paraphyses filiform, septate, hyaline to yellow brown, 4.5–8 µm wide at apex and 2.5–5 µm below. Ascospores ellipsoidal, hyaline, smooth, uniguttulate, 14.5–20 × 9.5–14.5 µm, Q = 1.25–1.6.

Additional specimens examined: China, Guangdong Province, Shaoguan City, Ruyuan Yao Autonomous County, Nanling National Nature Reserve, Xiaohuangshan, 24°53′47″ N, 113°1′8″ E, Alt. 1350 m, on the ground of broad-leaved forest, 15 June 2014, Tie-Zheng Wei 3917, HMAS 270958. Guizhou Province, Bijie City, Nayong County, Zuojiuga Yi and Miao Ethnic Town, 26°49′52″ N, 105°3′21″ E, on the ground of forest, 27 June 2018, Xing-Liang Wu 345, HMAS 290929. Yunnan Province, Kunming City, Anning City, Wenquan Town, Qiumuyuan, 24°59′7″ N, 102°26′58″ E, 20 July 1973, Qi-Ming Ma et al. 411, HMAS 186052; Honghe Hani and Yi Autonomous Prefecture, Pingbian Miao Autonomous County, Dawei Mountain National Nature Reserve, Shuiweicheng, 22°56′30″ N, 103°41′44″ E, Alt. 2100 m, 18 July 2005, Tie-Zheng Wei et al. 730, HMAS 131945. Zhejiang Province, Lishui City, Jingning She Autonomous County, Wangdongyang Nature Reserve, 27°42′32″ N, 119°36′59″ E, 4 June 2015, Mao-Qiang He ZRL20150069, HMAS 290930; *ibid.*, Yujikeng, 27°41′16″ N, 119°34′11″ E, 7 September 2019, Xin-Yu Zhu and Jia-Xin Li ZRL20191640, HMAS 290931.

Notes: This species was closely related to *H. vulgata* in all phylogenetic trees (Figure 1 and Figure S1–S4). It differs from the latter in 5 bp for Hsp90, 90 bp for ITS (73 bp in ITS1 and 17 bp in ITS2), 2 bp for LSU, and 3 bp for TEF. Morphological distinctions between them were hardly found (Table 3).

***Helvella vitrea*** X.C. Wang & W.Y. Zhuang, sp. nov. Figure 3L and Figure 5(30)

Fungal Names: FN571332

Etymology: The specific epithet refers to the semitransparent apothecia of this species.

Typification: China, Jiangsu Province, Nanjing City, Xuanwu District, Zijin Mountain, 32°4′17″ N, 118°50′57″ E, on soil, 29 May 2021, Zi-Han Zhang ZhangZH02, HMAS 290932, holotype.

Apothecia lobed, stipitate, 1–3.2 cm diam. And 1.8–4 cm high when dry; margin revolute or attached to the stipe; hymenium surface undulate-rugose, reddish brown to dark brown when dry; receptacle surface the same color with hymenium when dry; stipe surface sulcate, buff to yellow brown when dry, 1.6–3 × 0.25–1.8 cm when dry. Ectal excipulum not seen. Medullary excipulum of textura intricata, hyphae hyaline. Asci subcylindrical, tapering at base, eight-spored, 293–360 × 16–21 µm. Paraphyses filiform, septate, hyaline, 5–8 µm wide at apex and 2.5–4.5 µm below. Ascospores broad ellipsoidal, hyaline, smooth, uniguttulate, 14.5–18.5 × 12–14 µm, Q = 1.25.

Notes: The fungus formed a distinct lineage and was closely related to *H. huangii*, *H. parva*, *H. fulva*, *H. lobata*, *H. phlebophora*, and *H. phlebophoropsis* (Figure 1). Divergences in the molecular data were distinct enough to separate them at species level. It has much wider ascospores than related fungi (Table 3).

***Helvella vulgata*** X.C. Wang & W.Y. Zhuang, sp. nov. Figure 3G and Figure 5(14–15)

Fungal Names: FN571333

Etymology: The specific epithet refers to its common gross morphology shared with other *Helvella* species of this clade.

Typification: China, Hubei Province, Shennongjia Forestry District, Motianling, 31°30′55″ N, 110°35′1″ E, on the ground of broad-leaved forest, 26 August 1984, Jin-Xiu Tian 193, HMAS 53683, holotype.

Apothecia saddle-shaped, stipitate, 3 cm high when fresh, 0.4–1.1 cm diam. and 0.75–2.4 cm high when dry; margin revolute or attached to the stipe; hymenium surface undulate-rugose, yellow brown, orange brown, reddish brown, dark brown to black when dry; receptacle surface buff to yellow brown when dry, glabrous; stipe surface sulcate, buff, yellow brown to light brown when dry, 0.6–2 × 0.15–0.4 cm when dry. Ectal excipulum of textura angularis, cells hyaline to yellow brown, outer cells 17–37 × 6.5–17 µm. Medullary excipulum of textura intricata, hyphae hyaline. Asci subcylindrical, tapering at base, eight-spored, 240–306.5 × 12–17 µm. Paraphyses filiform, septate, hyaline to yellow brown, 4–6.5 µm wide at apex and 2.5–4 µm below. Ascospores ellipsoidal, hyaline, smooth, uniguttulate, 15–20 × 10.5–12 µm, Q = 1.55–1.6.

Additional specimens examined: China, Jilin Province, Changbai Mountain National Nature Reserve, on soil, 2 August 2008, Tai-Hui Li, HMIGD 25964; Yanbian Korean Autonomous Prefecture, Dunhua City, Dashitou Town, Alt. 670 m, on rotten wood in forest of *Quercus mongolica*, 15 September 1987, Jin-Zhong Cao 769, HMAS 85589.

Notes: This species was a sister of *H. varia* (Figure 1). Molecular differences were shown in the notes of the latter.

***Helvella yunnanensis*** X.C. Wang & W.Y. Zhuang, sp. nov. Figure 2T,U and Figure 5(29)

Fungal Names: FN571334

Etymology: The specific epithet refers to the type locality.

Typification: China, Yunnan Province, Kunming City, Panlong District, Yeya Lake, 25°7′22″ N, 102°51′36″ E, on soil, 25 September 2017, Huan-Di Zheng et al. 11785, HMAS 290933, holotype.

Apothecia saddle-shaped or lobed, stipitate, 1–2 cm diam. and 4–4.5 cm high when fresh, 0.4–0.9 cm diam. and 2.1–2.7 cm high when dry; margin revolute or attached to the stipe; hymenium surface undulate-rugose, light brown to dark brown when fresh, dark brown to black when dry; receptacle surface whitish or light brown when dry, glabrous; stipe surface sulcate, yellow brown to light brown when dry, 2–2.2 × 0.1–0.4 cm when dry. Ectal excipulum of textura angularis, cells hyaline, outer cells 16–36 × 6.5–16 µm. Medullary excipulum of textura intricata, hyphae hyaline. Asci subcylindrical, tapering at base, eight-spored, 280–320 × 13–20 µm. Paraphyses filiform, septate, hyaline to yellow brown, 5–8 µm wide at apex and 2.5–3 µm below. Ascospores ellipsoidal, hyaline, smooth, uniguttulate, 16–20 × 10.5–13 µm, Q = 1.5.

Additional specimen examined: China, Yunnan Province, Kunming City, Panlong District, Yeya Lake, 25°7′22″ N, 102°51′36″ E, on soil, 25 September 2017, Huan-Di Zheng et al. 11789, HMAS 290934.

Notes: This species has close relationships with *H. jizushanica* and *H. magna* (Figure 1), and molecular differences among them were discussed in the notes of the latter two fungi.

#### 3.2.2. New Chinese Records

***Helvella palustris*** Peck, Ann. Rep. N.Y. St. Mus. Nat. Hist. 33: 31, 1883.

Specimen examined: China, Jilin Province, Yanbian Korean Autonomous Prefecture, Antu County, Changbai Mountain National Nature Reserve, Alt. 1500 m, on rotten wood, 25 August 1960, Yu-Chuan Yang et al. 986, HMAS 30755.

Notes: *Helvella palustris* was originally described from New York, USA [4], and also reported from Norway, Finland, and Japan [20]. The Chinese collection is identical to the European material in the sequence of Hsp90.

***Helvella terricola*** Skrede & T. Schumach., Fungal Syst. Evol. 6: 91, 2020.

Specimen examined: China, Xinjiang Uygur Autonomous Region, Aksu Prefecture, Wensu County, Tuomuer Peak National Nature Reserve, Tailan River Valley, Alt. 2600 m, on the ground of forest, 24 July 1977, Hua-An Wen and Xiao-Lan Mao 158, HMAS 38355.

Notes: *Helvella terricola* was known only from Spain [22]. The Chinese collection extends its distribution to Asia. The Chinese collection is identical with the holotype in the sequence of Hsp90.

## 4. Discussion

A total of 101 specimens from four Chinese fungaria (HMAS, HMIGD, HMJAU, and CFSZ) and recent collections from 10 provinces (Beijing, Fujian, Guizhou, Jiangsu, Jilin, Shanxi, Sichuan, Tibet, Yunnan, and Zhejiang) were molecularly and morphologically examined in this study. Four loci were investigated, and 311 sequences were newly generated, including 101 for Hsp90, 82 for ITS, 69 for LSU, and 59 for TEF. A four-locus phylogeny of *Helvella lacunosa* clade was reconstructed, and 46 lineages were revealed in the tree. All these species occur in the Northern Hemisphere, and 25 of them are discovered in China. Nine species were previously known: *H. atra*, *H. cystidiata*, *H. lacunosa*, *H. palustris*, *H. philonotis*, *H. phlebophora*, *H. rugosa*, *H. sublactea*, and *H. Terricola*, while 16 species were determined as new to science and described and illustrated. Two new Chinese records, *H. palustris* and *H. terricola*, were also noted.

*Helvella rugosa* appears to be the most common species (17 specimens) of this clade in China with the widest distribution in the northeast, south, and southwest of the country (Table 1). The collections of the fungus formed a monophyletic clade in the phylogenies of ITS, LSU, and TEF. However, they were shown as polyphyletic in the Hsp90 tree and divided into four small groups (Figure S1). Although different intra-specific clustering of the examined collections existed in the phylogenies (Figures S1–S4), according to Genealogical Concordance Phylogenetic Species Recognition (GCPSR) [51], all of them should belong to the same species. Specimens from the same locality tended to cluster together (Figure 1), which might give the hint that they may be undergoing speciation. This might involve some underlying mechanism, e.g., incomplete lineage sorting.

Intra-specific variations in macro- and micro-morphology have been observed. For example, one specimen (Wu345) of *H. varia* has whitish apothecia when fresh (Figure 2J), while the others (ZRL20150069 and ZRL20191640) are brown to nearly black (Figure 2K,L), which might influenced by fruitbody age, the ecological niches of a collection, as well as the degree of exposure to light. The collections of *H. magna* also exhibited apothecial color variations (Figure 2D–F). Additionally, apothecial size is also variable within individual species: some collections (HMAS 60679, HMAS 61724, and HMAS 66121, all from Gansu Province) of *H. magna* possess large apothecia (up to 5–6 cm high when dry) and inflated stipes (up to 1.7–2.4 cm diam. when dry, Figure 3F), whereas the others have smaller apothecia (usually less than 2 cm high when dry) and thinner stipes (no more than 0.5 cm diam. when dry); nutrition or texture of the substrates might end up with size changes of apothecia. Microscopically, different lengths of asci were found in *H. austrooccidentalis*, *H. magna,* and *H. varia* (Table 3); the width of the paraphysis apices among collections varied in *H. austrooccidentalis*, *H. cystidiata*, *H. magna,* and *H. plateata*; the color of paraphyses varied in *H. varia* and *H. vulgata*; and variations of the shape of ascospores can be seen in *H. magna* and *H. varia* (Figure 5 and Table 3). These noticeable morphological differences make identifications solely based on morphology unreliable.

Our understanding of the *Helvella lacunosa* clade in China is significantly renewed through this work. *Helvella pseudolacunosa* was proved to be a later synonym of *H. lacunosa* (Figures S2 and S3). Additionally, the previous records of *H. fusca*, *H. helvellula*, *H. lactea,* and *H. sulcata* in China were based on misidentifications and should be excluded from Chinese fungus flora. The unexpectedly high biodiversity of the lacunosa clade suggests that species diversity of macrofungi in Pezizales may have been underestimated. Further large-scale investigations are desperately needed to examine unexplored pezizalean fungi.

## Figures and Tables

**Table 2:** Detailed characteristics of the phylogenetic analysis datasets.

Locus	No. of Seq.	Length of Alignment (bp)	No. of Variable Sites	No. of Parsimony-Informative Sites	Model for BI
Hsp90 + ITS + LSU + TEF	135	2652	857	709	TVM + I + G
Hsp90	135	240	68	60	
ITS	100	975	491	443	
LSU	105	874	154	106	
TEF	72	567	173	141	

Full name of the used model: TVM + I + G (transversion model with invariable sites and gamma distribution).

**Table 3:** Morphological comparisons among species of *Helvella lacunosa* clade.

Species	Specimen	Asci (µm)	Paraphyses Apex Width (µm)	Paraphyses Apex Color	Paraphyses Width (µm)	Paraphyses Color	Outer Cells (µm)	Ascospores (µm)	Mean of Q
*H. austrooccidentalis*	11223, holotype	260–333 × 14.5–21	6.5–13	yellow brown	2.5–4	hyaline to yellow brown	26.5–46.5 × 9–20	17–21 × 10.5–14.5	1.5
	ZRL20200655	313–400 × 13–17	3–6	yellow brown	2.5–3	yellow brown	12–26.5 × 6–10.5	16–22 × 10.5–12.5	1.65
*H. borealis*	3568, holotype	240–280 × 12–14.5	6.5–10.5	hyaline	3.5–5	hyaline	17–73 × 6.5–37	14.5–16 × 9–10.5	1.55
*H. cystidiata*	HKAS 78941, holotype	200–330 × 13–19	4–8	hyaline	3–5	hyaline	10–35 × 8–20	15–18.5 × 9–12	1.6
	HMAS 275836	326.5–346.5 × 14.5–20	4–8	hyaline to yellow brown	2.5–4	hyaline to yellow brown		17.5–20 × 10.5–13	1.6
	HMJAU 150	326.5–386.5 × 13.5–16	8–16	hyaline	2.5–4	hyaline to yellow brown	24–66.5 × 9.5–21	17–22 × 10.5–12.5	1.7
*H. fulva*	10867, holotype	280–346.5 × 13–21	5–9	hyaline to yellow brown	2.5–4	hyaline to yellow brown	26.5–33 × 8–13	17–20 × 10.5–12.5	1.6
*H. huangii*	HMAS 45031, holotype	213.5–280 × 13.5–18.5	5.5–8	hyaline	3.5–5.5	hyaline to yellow brown	26.5–36 × 8–18.5	17–20 × 11–13	1.5
	HMJAU 3488	246.5–320 × 14.5–18.5	4–5	hyaline	4–5	hyaline to yellow brown			
	CFSZ 2652	253–300 × 13.5–18.5	4.5–6.5	hyaline to yellow brown	2.5–4	hyaline to yellow brown	20–40 × 9–12	14.5–20 × 10.5–12.5	1.5
*H. jizushanica*	11567, holotype	246.5–320 × 13–18.5	6–8.5	hyaline	2.5	hyaline to yellow brown	17–46.5 × 10.5–29	16–21 × 10.5–12.5	1.6
*H. liui*	HMAS 85725, holotype	266.5–333 × 16–18.5	5–6.5	hyaline	3–4	hyaline	20–33 × 5–10.5	17–20 × 11–13	1.5
*H. lobata*	HaiY01, holotype	220–320 × 12–20	5–6.5	hyaline	2.5–4	hyaline to yellow brown	24–46.5 × 8–18.5	16–20 × 12–14.5	1.35
*H. magna*	10861	326.5–413 × 13–17	6.5–13	hyaline to yellow brown	2.5–6	hyaline to yellow brown	20–88 × 8–30.5	20–22.5 × 12–14.5	1.6
	11790	306.5–340 × 13–18.5	3–4	hyaline	2.5–3	hyaline			
	HMAS 60679, holotype	233–320 × 14.5–20	8–10	hyaline to yellow brown	4–5	hyaline to yellow brown	26.5–44 × 13–21	17–22.5 × 11–13	1.65
	HMAS 70345	280–320 × 13–18.5	5–8	hyaline to yellow brown	2.5–4	hyaline to yellow brown		15–21 × 10.5–16	1.35
*H. parva*	11559, holotype	226.5–266.5 × 13–17	6.5	hyaline	3.5–4	hyaline to yellow brown	20–53 × 6.5–20	17–18.5 × 10.5–12	1.6
*H. phlebophoropsis*	HMAS 85654, holotype	240–280 × 13–20	5–8	hyaline to yellow brown	2.5–4	hyaline to yellow brown	33–42.5 × 5–12	14.5–20 × 10.5–13	1.5
	HMAS 30757	246.5–266.5 × 13–20	4–6.5		4	hyaline to yellow brown	24–25 × 8–9	16.5–21 × 10.5–13	1.6
*H. plateata*	HMAS 270642, holotype	246.5–426.5 × 14.5–20	4–6.5	hyaline to yellow brown	2.5–4	hyaline to yellow brown	24–33 × 6.5–12	20–22.5 × 11–13	1.75
	11595	260–333 × 13–20	6.5–9	light brown to brown	2.5–5	light brown	26.5–46.5 × 9–21	18.5–23 × 11–12.5	1.75
	ZRL20201123	206.5–300 × 15–20	6.5–10.5	light brown to brown	3–5	light brown to brown	18.5–60 × 9–24	16–21 × 9–12.5	1.7
*H. ravida*	10682, holotype	240–333 × 14.5–24	8–13	hyaline	4–4.5	hyaline	17–57 × 8–25	16–18.5 × 10.5–13	1.5
	HMAS 61920	253 × 14.5				hyaline to yellow brown		16–20 × 10.5–13	1.5
*H. varia*	HMAS 131945	230–300 × 13.5–22.5	5–8	hyaline to yellow brown	2.5–5	hyaline to yellow brown	14.5–40 × 9–21.5	16–20 × 10.5–13.5	1.5
	HMAS 270932, holotype	240–306.5 × 14.5–17	4.5–8	hyaline to yellow brown	4	yellow brown		14.5–18.5 × 12–14.5	1.25
	Wu 345	193–246.5 × 14.5–18.5	5.5–8	hyaline	3.5–4.5	hyaline	21–46.5 × 9–21	14.5–17.5 × 9.5–12	1.5
	ZRL20150069		5.5–6.5	hyaline	2.5–4	yellow brown	17.5–41.5 × 10.5–20	16–20 × 10.5–12	1.6
*H. vitrea*	ZhangZH02, holotype	293–360 × 16–21	5–8	hyaline	2.5–4.5	hyaline		14.5–18.5 × 12–14	1.25
*H. vulgata*	HMAS 53683, holotype	260–293 × 13–16	4.5–6.5	hyaline to yellow brown	2.5–4	hyaline to yellow brown	17–37 × 6.5–17	16–20 × 10.5–12	1.6
	HMIGD 25964	240–306.5 × 12–17	4–6.5	hyaline	2.5–3	hyaline		15–20 × 10.5–12	1.55
*H. yunnanensis*	11785	280–320 × 13–20	5–8	hyaline	2.5–3	hyaline to yellow brown	16–36 × 6.5–16	16–20 × 10.5–13	1.5

## Data Availability

All sequence data generated for this study (Table 1) can be accessed via GenBank: https://www.ncbi.nlm.nih.gov/genbank/ (accessed on 8 March 2023).

## Supplementary Materials

The following supporting information can be downloaded at: https://www.mdpi.com/article/10.3390/jof9070697/s1, Figure S1: Maximum-likelihood phylogenetic tree of Helvella lacunosa clade inferred from combined Hsp90 dataset. Bootstrap values ≥ 70% (left) are indicated at nodes. Asterisk denotes 100% bootstrap; Figure S2: Maximum-likelihood phylogenetic tree of Helvella lacunosa clade inferred from combined ITS dataset. Bootstrap values ≥ 70% (left) are indicated at nodes. Asterisk denotes 100% bootstrap; Figure S3: Maximum-likelihood phylogenetic tree of Helvella lacunosa clade inferred from combined LSU dataset. Bootstrap values ≥ 70% (left) are indicated at nodes. Asterisk denotes 100% bootstrap; Figure S4: Maximum-likelihood phylogenetic tree of Helvella lacunosa clade inferred from combined TEF1 dataset. Bootstrap values ≥ 70% (left) are indicated at nodes. Asterisk denotes 100% bootstrap.

## References

[1] Khalid M., Tan H., Ali M., Rehman A., Liu X., Su L., Saeed Ur R., Zhao C., Li X., Hui N. (2022). Karst rocky desertification diverged the soil residing and the active ectomycorrhizal fungal communities thereby fostering distinctive extramatrical mycelia. Sci. Total Environ..

[2] Hwang J., Zhao Q., Yang Z.L., Wang Z., Townsend J.P. (2015). Solving the ecological puzzle of mycorrhizal associations using data from annotated collections and environmental samples—An example of saddle fungi. Environ. Microbiol. Rep..

[3] Dissing H. (1966). The Genus Helvella in Europe, with Special Emphasis on the Species Found in Norden.

[4] Weber N.S. (1972). The genus *Helvella* in Michigan. Mich. Bot..

[5] Häffner J. (1987). Die Gattung *Helvella*. Morphologie und Taxonomie. Beih. Z. Mykol..

[6] O’Donnell K., Cigelnik E., Weber N.S., Trappe J.M. (1997). Phylogenetic relationships among ascomycetous truffles and the true and false morels inferred from 18S and 28S ribosomal DNA sequence analysis. Mycologia.

[7] Harrington F.A., Pfister D.H., Potter D., Donoghue M.J. (1999). Phylogenetic studies within the Pezizales. I. 18S rRNA sequence data and classification. Mycologia.

[8] Landvik S., Kristiansen R., Schumacher T. (1999). *Pindara*: A miniature Helvella. Mycologia.

[9] Percudani R., Trevisi A., Zambonelli A., Ottonello S. (1999). Molecular phylogeny of truffles (Pezizales: Terfeziaceae, Tuberaceae) derived from nuclear rDNA sequence analysis. Mol. Phylogenet. Evol..

[10] Hansen K., Pfister D.H. (2006). Systematics of the Pezizomycetes—The operculate discomycetes. Mycologia.

[11] Tedersoo L., Hansen K., Perry B.A., Kjoller R. (2006). Molecular and morphological diversity of pezizalean ectomycorrhiza. New Phytol..

[12] Laessoe T., Hansen K. (2007). Truffle trouble: What happened to the Tuberales?. Mycol. Res..

[13] Perry B.A., Hansen K., Pfister D.H. (2007). A phylogenetic overview of the family Pyronemataceae (Ascomycota, Pezizales). Mycol. Res..

[14] Spatafora J.W., Sung G.H., Johnson D., Hesse C., O’Rourke B., Serdani M., Spotts R., Lutzoni F., Hofstetter V., Miadlikowska J. (2006). A five-gene phylogeny of Pezizomycotina. Mycologia.

[15] Taskin H., Buyukalaca S., Dogan H.H., Rehner S.A., O’Donnell K. (2010). A multigene molecular phylogenetic assessment of true morels (*Morchella*) in Turkey. Fung. Genet. Biol..

[16] O’Donnell K., Rooney A.P., Mills G.L., Kuo M., Weber N.S., Rehner S.A. (2011). Phylogeny and historical biogeography of true morels (*Morchella*) reveals an early Cretaceous origin and high continental endemism and provincialism in the Holarctic. Fungal Genet. Biol..

[17] Bonito G., Smith M.E., Nowak M., Healy R.A., Guevara G., Cazares E., Kinoshita A., Nouhra E.R., Dominguez L.S., Tedersoo L. (2013). Historical biogeography and diversification of truffles in the Tuberaceae and their newly identified southern hemisphere sister lineage. PLoS ONE.

[18] Nguyen N.H., Landeros F., Garibay-Orijel R., Hansen K., Vellinga E.C. (2013). The *Helvella lacunosa* species complex in western North America: Cryptic species, misapplied names and parasites. Mycologia.

[19] Landeros F., Iturriaga T., Rodríguez A., Vargas-Amado G., Guzmán-Dávalos L. (2015). Advances in the phylogeny of *Helvella* (Fungi: Ascomycota), inferred from nuclear ribosomal LSU sequences and morphological data. Rev. Mex. Biodivers..

[20] Skrede I., Carlsen T., Schumacher T. (2017). A synopsis of the saddle fungi (*Helvella*: Ascomycota) in Europe—Species delimitation, taxonomy and typification. Persoonia.

[21] Wang X.C., Liu T.Z., Chen S.L., Li Y., Zhuang W.Y. (2019). A four-locus phylogeny of rib-stiped cupulate species of *Helvella* (Helvellaceae, Pezizales) with discovery of three new species. MycoKeys.

[22] Skrede I., Gonzalvo L.B., Mathiesen C., Schumacher T. (2020). The genera *Helvella* and *Dissingia* (Ascomycota: Pezizomycetes) in Europe—Notes on species from Spain. Fung. Syst. Evol..

[23] Landeros F., Ferrusca-Rico F.M., Guzmán-Dávalos L., Esquivel-Naranjo E.U., Matías-Ferrer N., Burrola-Aguilar C., Viurcos-Martínez G.A., Garibay-Orijel R. (2021). *Helvella jocatoi* sp. nov. (Pezizales, Ascomycota), a new species from *H. lacunosa* complex with cultural importance in central Mexico Abies religiosa forests. Phytotaxa.

[24] Xu R.J., Li L., Zhao Q. (2022). *Helvella cystidiata* sp. nov. (Helvellaceae, Ascomycota) from Tibetan Plateau, China. Phytotaxa.

[25] Teng S.C. (1963). Fungi of China.

[26] Tai F.L. (1979). Sylloge Fungorum Sinicorum.

[27] Liu B., Cao J.Z. (1988). Some new species and new records of the genus *Helvella* from China (I). Acta Mycol. Sin..

[28] Zhuang W.Y., Wang Z. (1998). Some new species and new records of Discomycetes in China. VIII. Mycotaxon.

[29] Xu A.S. (2002). Notes on *Helvella* in Xizang. Mycosystema.

[30] Ariyawansa H.A., Hyde K.D., Jayasiri S.C., Buyck B., Chethana K.W.T., Dai D.Q., Dai Y.C., Daranagama D.A., Jayawardena R.S., Lücking R. (2015). Fungal diversity notes 111–252—Taxonomic and phylogenetic contributions to fungal taxa. Fungal Divers..

[31] Wang M., Zhao Y.C., Zhao Q., Zhou D.Q. (2016). *Helvella sublactea* sp. nov. (Helvellaceae) from southwestern China. Phytotaxa.

[32] Wang X.C., Zhuang W.Y. (2019). A three-locus phylogeny of *Gyromitra* (Discinaceae, Pezizales) and discovery of two cryptic species. Mycologia.

[33] White T.J., Bruns T.D., Lee S.B., Taylor J.W., Innis M.A., Gelfand D.H., Sninsky J.J., White T.J. (1990). Amplification and direct sequencing of fungal ribosomal RNA genes for phylogenetics. PCR Protocols: A Guide to Methods and Applications.

[34] Vilgalys R., Hester M. (1990). Rapid genetic identification and mapping of enzymatically amplified ribosomal DNA from several *Cryptococcus* species. J. Bacteriol..

[35] Rehner S.A., Buckley E. (2005). A *Beauveria* phylogeny inferred from nuclear ITS and EF1-a sequences: Evidence for cryptic diversification and links to *Cordyceps* teleomorphs. Mycologia.

[36] Katoh K., Standley D.M. (2013). MAFFT multiple sequence alignment software version 7: Improvements in performance and usability. Mol. Biol. Evol..

[37] Hall T.A. (1999). BioEdit: A user-friendly biological sequence alignment editor and analysis program for Windows 95/98/NT. Nucleic Acids Symp. Ser..

[38] Tamura K., Stecher G., Peterson D., Filipski A., Kumar S. (2013). MEGA6: Molecular Evolutionary Genetics Analysis version 6.0. Mol. Biol. Evol..

[39] Stamatakis A. (2006). RAxML-VI-HPC: Maximum likelihood-based phylogenetic analyses with thousands of taxa and mixed models. Bioinformatics.

[40] Miller M.A., Pfeiffer W., Schwartz T. Creating the CIPRES Science Gateway for inference of large phylogenetic trees. Proceedings of the Gateway Computing Environments Workshop (GCE).

[41] Ronquist F., Teslenko M., van der Mark P., Ayres D.L., Darling A., Hohna S., Larget B., Liu L., Suchard M.A., Huelsenbeck J.P. (2012). MrBayes 3.2: Efficient Bayesian phylogenetic inference and model choice across a large model space. Syst. Biol..

[42] Posada D., Crandall K.A. (1998). MODELTEST: Testing the model of DNA substitution. Bioinformatics.

[43] Løken S.B., Skrede I., Schumacher T. (2020). The *Helvella corium* species aggregate in Nordic countries—Phylogeny and species delimitation. Fungal Syst. Evol..

[44] Hansen K., Schumacher T., Skrede I., Huhtinen S., Wang X.-H. (2019). *Pindara* revisited—Evolution and generic limits in Helvellaceae. Persoonia.

[45] Zhao Q., Sulayman M., Zhu X.T., Zhao Y.C., Yang Z.L., Hyde K.D. (2016). Species clarification of the culinary Bachu mushroom in western China. Mycologia.

[46] Wei S.P., Song Y.J., Jia L.M., Yuan Z. (2018). Diversity of ectomycorrhizal fungi associated with *Quercus variabilis* in gneissose area of Taihang Mountains. Mycosystema.

[47] Zhao Q., Tolgor B., Zhao Y., Yang Z.L., Hyde K.D. (2015). Species diversity within the *Helvella crispa* group (Ascomycota: Helvellaceae) in China. Phytotaxa.

[48] Ekanayaka A.H., Hyde K.D., Jones E.B.G., Zhao Q. (2018). Taxonomy and phylogeny of operculate discomycetes: Pezizomycetes. Fungal Divers..

[49] Hansen K., Perry B.A., Dranginis A.W., Pfister D.H. (2013). A phylogeny of the highly diverse cup-fungus family Pyronemataceae (Pezizomycetes, Ascomycota) clarifies relationships and evolution of selected life history traits. Mol. Phylogenet. Evol..

[50] Abbott S.P., Currah R.S. (1997). The Hevellaceae: Systematic revision and occurrence in northern and northwestern North America. Mycotaxon.

[51] Taylor J.W., Jacobson D.J., Kroken S., Kasuga T., Geiser D.M., Hibbett D.S., Fisher M.C. (2000). Phylogenetic species recognition and species concepts in fungi. Fungal Genet. Biol..

