# Supplementary material for: Species Diversity of Helvella lacunosa Clade (Pezizales, Ascomycota) in China and Description of Sixteen New Species
Source: J Fungi (Basel). 2023 Jun 23;9(7):697. doi: 10.3390/jof9070697 (PMC10381826; doi:10.3390/jof9070697)
Supplement: Supplementary file 1 [file jof-09-00697-s001.zip › Supplementary Legends.pdf]

Legends for Supplementary Figures

Figure S1. Maximum-likelihood phylogenetic tree of *Helvella lacunosa* clade inferred from combined Hsp90 dataset. Bootstrap values ≥70% (left) are indicated at nodes. Asterisk denotes 100% bootstrap.

Figure S2. Maximum-likelihood phylogenetic tree of *Helvella lacunosa* clade inferred from combined ITS dataset. Bootstrap values ≥70% (left) are indicated at nodes. Asterisk denotes 100% bootstrap.

Figure S3. Maximum-likelihood phylogenetic tree of *Helvella lacunosa* clade inferred from combined LSU dataset. Bootstrap values ≥70% (left) are indicated at nodes. Asterisk denotes 100% bootstrap.

Figure S4. Maximum-likelihood phylogenetic tree of *Helvella lacunosa* clade inferred from combined TEF1 dataset. Bootstrap values ≥70% (left) are indicated at nodes. Asterisk denotes 100% bootstrap.
