# Supplementary figures and images for: Species Diversity of Helvella lacunosa Clade (Pezizales, Ascomycota) in China and Description of Sixteen New Species
Source: J Fungi (Basel). 2023 Jun 23;9(7):697. doi: 10.3390/jof9070697 (PMC10381826; doi:10.3390/jof9070697)

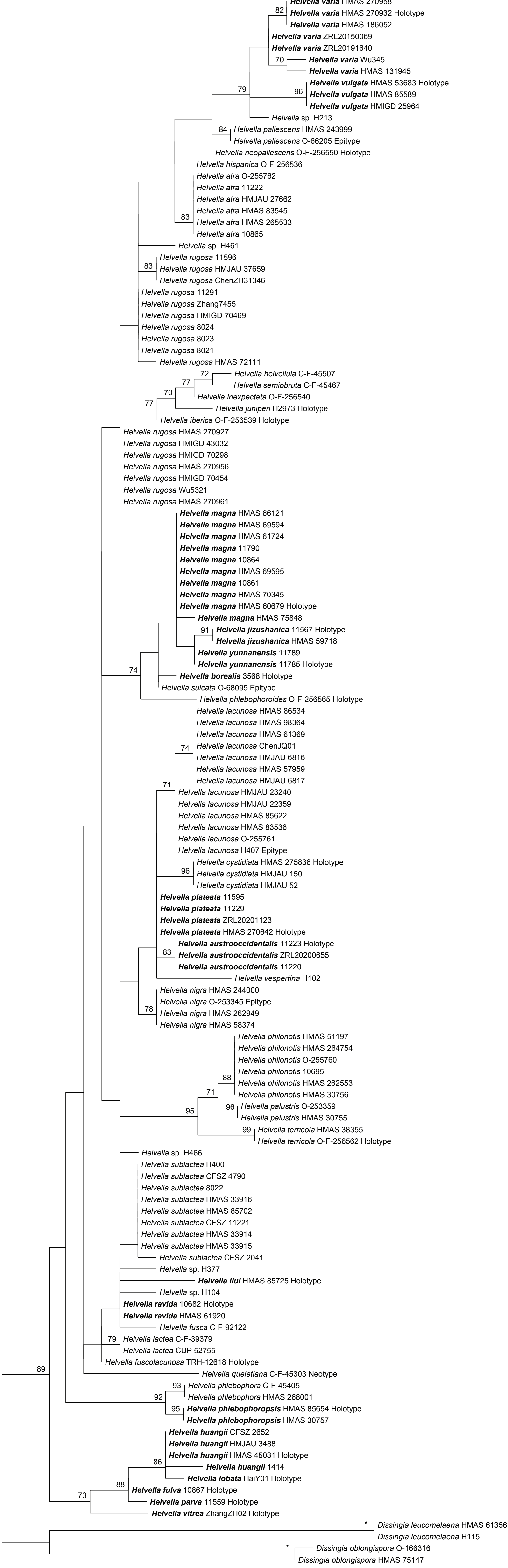

Supplement: Supplementary file 1 [file jof-09-00697-s001.zip › FigureS1 Hsp90.pdf]

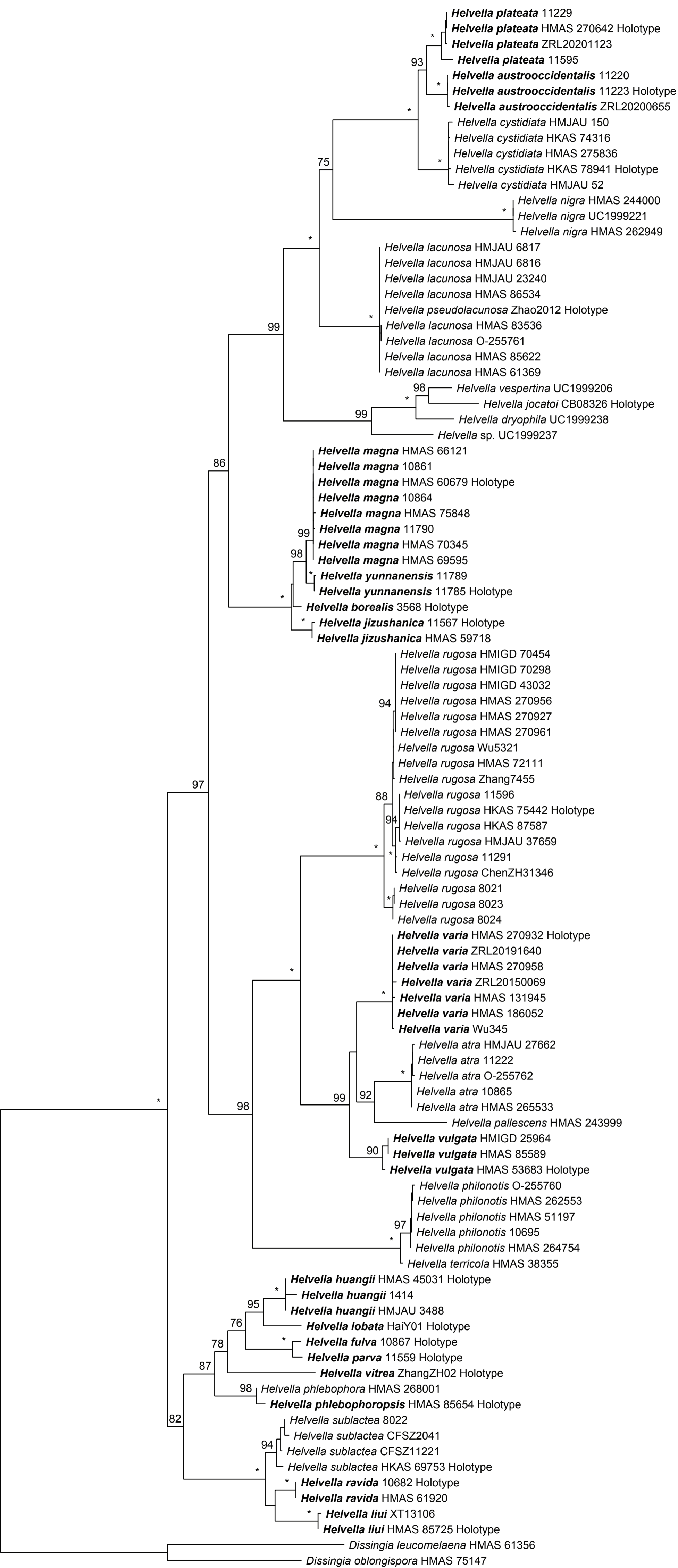

Supplement: Supplementary file 1 [file jof-09-00697-s001.zip › FigureS2 ITS.pdf]

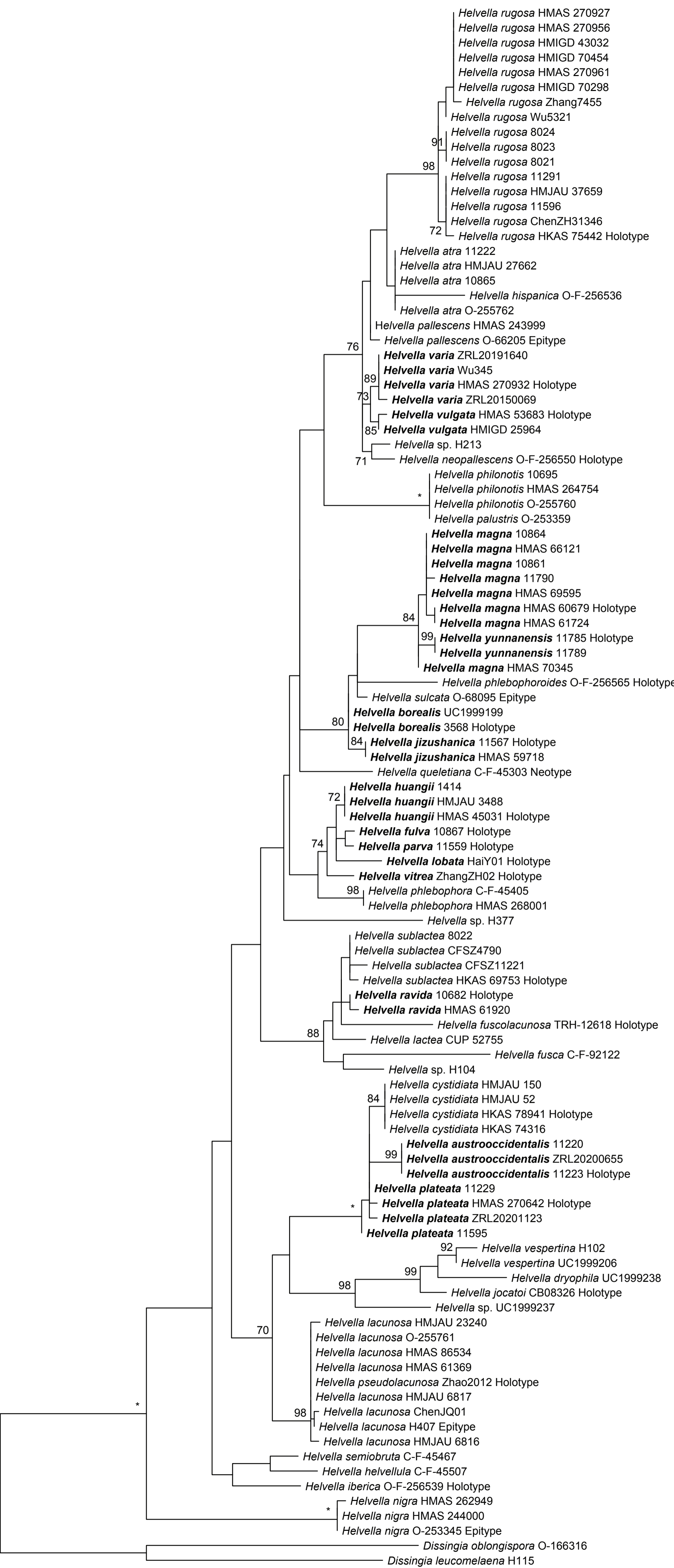

Supplement: Supplementary file 1 [file jof-09-00697-s001.zip › FigureS3 LSU.pdf]

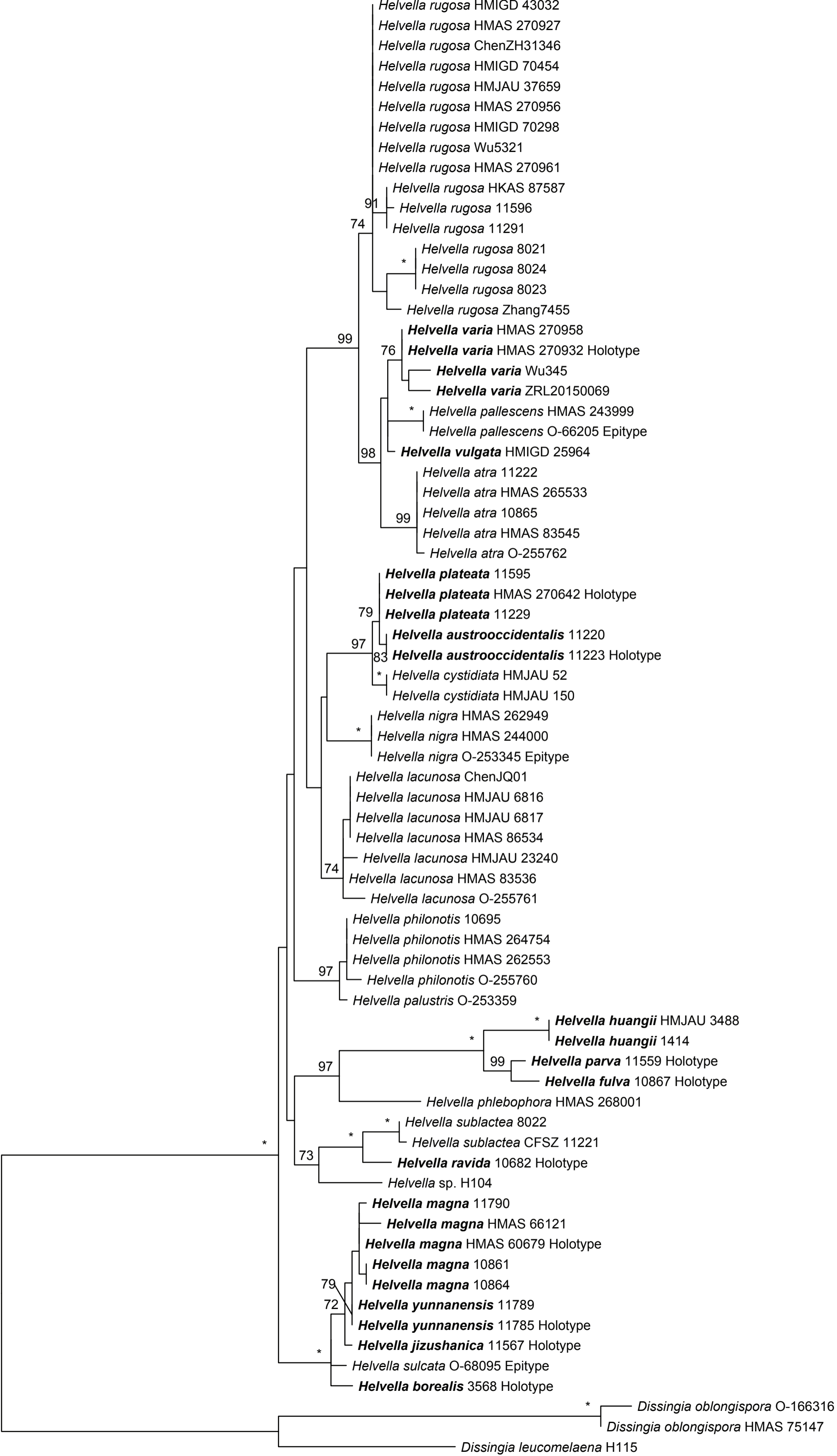

Supplement: Supplementary file 1 [file jof-09-00697-s001.zip › FigureS4 TEF.pdf]
